# Allogeneic stem-cell transplantation for multiple myeloma: a systematic review and meta-analysis from 2007 to 2017

**DOI:** 10.1186/s12935-018-0553-8

**Published:** 2018-04-23

**Authors:** Xuejiao Yin, Liang Tang, Fengjuan Fan, Qinyue Jiang, Chunyan Sun, Yu Hu

**Affiliations:** 1 Institute of Hematology, Union Hospital, Tongji Medical College, Huazhong University of Science and Technology, Jiefang Dadao, Wuhan, 430022 China; 2 Collaborative Innovation Center of Hematology, Huazhong University of Science and Technology, Jiefang Dadao, Wuhan, 430022 China

**Keywords:** Multiple myeloma, Allogeneic transplantation, OS, PFS, RR, Death rate, TRM, GVHD

## Abstract

**Background:**

Despite recent advances, multiple myeloma (MM) remains incurable. However, the appearance of allogeneic stem cell transplantation (allo-SCT) through graft-versus-myeloma effect provides a potential way to cure MM to some degree. This systematic review aimed to evaluate the outcome of patients receiving allo-SCT and identified a series of prognostic factors that may affect the outcome of allo-SCT.

**Patients/methods:**

We systematically searched PubMed, Embase, and the Cochrane Library from 2007.01.01 to 2017.05.03 using the keywords ‘allogeneic’ and ‘myeloma’.

**Results:**

A total of 61 clinical trials involving 8698 adult patients were included. The pooled estimates (95% CI) for overall survival (OS) at 1, 2, 3 and 5 years were 70 (95% CI 56–84%), 62 (95% CI 53–71%), 52 (95% CI 44–61%), and 46 (95% CI 40–52%), respectively; for progression-free survival were 51 (95% CI 38–64%), 40 (95% CI 32–48%), 34 (95% CI 27–41%), and 27 (95% CI 23–31%), respectively; and for treatment-related mortality (TRM) were 18 (95% CI 14–21%), 21 (95% CI 17–25%), 20 (95% CI 13–26%), and 27 (95% CI 21–33%), respectively. Additionally, the pooled 100-day TRM was 12 (95% CI 5–18%). The incidences of grades II–IV acute graft-versus-host disease (GVHD) and chronic GVHD were 34 (95% CI 30–37%) and 51 (95% CI 46–56%), respectively. The incidences of relapse rate (RR) and death rate were 50 (95% CI 45–55%) and 51 (95% CI 45–57%), respectively. Importantly, disease progression was the most major cause of death (48%), followed by TRM (44%). The results failed to show an apparent benefit of allo-SCT for standard risk patients, compared with tandem auto-SCT. In contrast, all 14 trials in our study showed that patients with high cytogenetic risk after allo-SCT had similar OS and PFS compared to those with standard risk, suggesting that allo-SCT may overcome the adverse prognosis of high cytogenetic risk.

**Conclusion:**

Due to the lack of consistent survival benefit, allo-SCT should not be considered as a standard of care for newly diagnosed and relapsed standard-risk MM patients. However, for patients with high-risk MM who have a poor long-term prognosis, allo-SCT may be a strong consideration in their initial course of therapy or in first relapse after chemotherapy, when the risk of disease progression may outweigh the transplant-related risks. A large number of prospective randomized controlled trials were needed to prove the benefits of these therapeutic options.

**Electronic supplementary material:**

The online version of this article (10.1186/s12935-018-0553-8) contains supplementary material, which is available to authorized users.

## Background

Multiple myeloma (MM) is an incurable clonal plasma cell hematologic malignancy. It is usually a disease of the elderly and its’ median age at diagnosis is 65 years [1]. Modern therapy for MM includes corticosteroids, immune-modulatory drugs (IMiDs) (thalidomide, lenalidomide, pomalidomide) [2], proteasome inhibitors (bortezomib, carfilzomib) [3], compounds targeting specific molecules and monoclonal antibodies. Besides, autologous stem cell transplantation (auto-SCT) in combination with high-dose chemotherapy could be considered as a frontline strategy for younger MM patients [4]. Although these therapies dramatically increased patients’ response rate and survival rate [5], most patients could not maintain a sustained complete remission and relapse ultimately. Allogeneic SCT (allo-SCT) through graft-versus-myeloma (GvM) effect was gradually emerging as a potential way to cure MM [6]. However, allo-SCT couldn’t be widely used to treat MM patients [7] due to unusable donors, high risk of treatment related mortality (TRM) and the occurrence of GVHD during several decades. Major transplant innovations and new technologies emerged in 2000. The European Group for Blood and Marrow Transplantation (EBMT) registry reported that the usage rates of PBSC grew from 66 to 91%, while RIC/NMA from 37 to 75% during the time of 1998–1999 to 2002–2003 [8]. Moreover, since 2000, novel drugs have been applied in different phases of allo-SCT, such as conditioning regimen and post-transplantation therapy, the introduction of which brought additional clinical benefits, improved responses rates and made the transplant safer. Additionally, with the increasing awareness of the risk stratification of MM and the improvement of detection techniques, it is now possible to identify high-risk MM patients more quickly and accurately [9]. High cytogenetic risk, as a poor prognostic factor, may encourage these patients receiving transplants earlier. Indeed, Nivison-Smith et al. reported the shorter length of time between diagnosis and transplant was a prognostic factor index for both improved OS and PFS [10]. Furthermore, the occurrence rate of TRM and GVHD has been reduced through advanced maintenance strategies, better supportive care, more suitable patient selection and strategies for GVHD prophylaxis. Therefore, allo-SCT maybe well tolerated and become an effective way to cure MM in the future. Till now, the indications which guided clinicians to apply allo-SCT to clinical practice are mainly derived from large registry and single-center retrospective studies. Thus we performed a systematic literature review and meta-analysis to evaluate the efficacy and safety of allo-SCT for MM during the last 10 years.

## Methods

### Search strategy

We searched MEDLINE, Embase, and the Cochrane Library database. We used ‘allogeneic AND myeloma’ for Medline and the Cochrane Library search, and the following terms for Embase search: ‘allogeneic’.mp. AND ‘myeloma’.mp. Adults, humans and English language articles were limited to our search. We conducted our search from 2007.01.01 to the 2017.05.03.

### Inclusion criteria, exclusion criteria

Inclusion criteria: (1) studies involving patients with multiple myeloma, (2) treatment with allo-SCT, (3) a sample size ≥ 5, (4) reported in English, (5) the date of publication from 2007.01.01 to the 2017.05.03, (6) the species of human, adults.

Exclusion criteria: (1) the use of cord blood as the stem cell source, (2) the inclusion of patients with various hematological malignancies without a separate description of the results of MM patients, (3) the use of a wide variety of transplant strategies rather than one clearly defined strategy that was similar for each patient, (4) lack of outcome data.

### Study selection and data extraction

We only considered full-text articles. The titles and abstracts of the remaining articles were screened by the following exclusion criteria: reviews, meta-analyses, editorials, conference proceedings, no primary or secondary endpoints reported and commentaries. Study selection, quality assessment and data extraction were conducted by two reviewers independently using standardized forms. If there were disagreements, third investigator would adjudicate.

### Study quality assessment

We followed 5 items to evaluate study quality: (1) conditioning regimens, (2) stem cell source, (3) donor, (4) GvHD prophylaxis regimen, and (5) disease status before allo-SCT. When articles provided one corresponding item, 1 was given to the study or otherwise 0. Only studies received 5 scores were deem as good quality, 4 scores were moderate quality and 3 scores were low quality.

### Outcome indicator

The primary endpoints were the 1-, 2-, 3- and 5-years overall survival (OS) and progression-free survival (PFS)/disease-free survival (DFS). Secondary endpoints were graft-versus-host disease (GvHD), relapse rate (RR), death rates and the 100-day, 1-, 2-, 3- and 5-year treatment-related mortality (TRM).

### Heterogeneity analyses and subgroup analyses

Study heterogeneity was assessed with the Cochran Q test, and the I^2^ statistic was used to quantify it. If the p value of Cochran’s Q test was < 0.1 and I^2^ statistic was > 50%, it indicated the substantial heterogeneity was existent. We used a random-effect model to pool the data. If we found substantial heterogeneity, subgroup analyses were performed to explore the causes. Specifically, the subgroups transplantation period 1990s versus 2000s were considered. We identified the following prognostic factors that may affect the outcome of allo-SCT: cytogenetic risk (high-risk versus non-high-risk), remission status at the time of transplantation and post transplantation (CR versus non-CR), source of the transplanted stem cell (peripheral blood stem cell versus bone marrow).

### Sensitivity analyses

Sensitivity analyses were performed to test the robustness of the results.

### Publication bias

Publication bias was assessed using funnel plots, begg and Egger test. p values < 0.05 were considered statistically significant. Analyses were performed with Review Manager (version 5.1) and Stata SE/MP 11.0.

## Result

Our initial search yielded 3144 articles. After screening, 393 duplicating studies were removed, and 2654 studies were excluded based on titles and abstracts. A further 36 studies were excluded for not fulfilling the inclusion criteria. Finally, a total of 61 citations with 8698 eligible patients were included in the meta-analysis. The sample size varied from 7 to 1667. Follow-up period ranged from 1 to 217.2 months. Patients’ age ranged between 21 and 77 years. According to quality assessment scores, 29 studies scored 5, 25 scored 4, and 7 scored 3.

### OS

Most studies reported the results of OS [10–48]. The pooled estimates (95% CI) of OS at 1, 2, 3 and 5 years were 70 (95% CI 56–84%), 62 (95% CI 53–71%), 52 (95% CI 44–61%), and 46 (95% CI 40–52%), respectively. High heterogeneity was found in these studies (p = 0, I^2^ = 96.9, 89.8, 95.6, 93.9%, respectively). Subgroup analysis demonstrated a statistically significant benefit of OS in patients who underwent planned autologous transplantation before allo-SCT compared with direct allo-SCT (RR = 1.28, 95% CI 1.11–1.49) [17, 26]. There was no difference in OS between autologous stem cell transplantation followed by allogeneic stem cell transplantation (auto-allo-SCT) and tandem autologous stem cell transplantation (tandem-auto-SCT) (RR = 0.91, 95% CI 0.77–1.06) [16, 18, 19, 42, 46, 49].

During the past decade, myeloablative conditioning (MA) has been largely abandoned due to the high treatment related mortality (TRM). However, reduced-intensity conditioning (RIC) or non-myeloablative conditioning (NMA) regimens have high risk of relapse. To estimate whether the widespread adoption of RIC/NMA regimens could bring about better OS, we compared patients receiving allo-SCT with RIC/NMA regimen with those receiving MA regimens in OS. No evidence was found that RIC/NMA regimens improved OS compared to MA regimens (RR = 0.88, 95% CI 0.74–1.05) [10, 13, 25, 29]. These results suggested that different conditioning regimens didn’t affect OS.

Some studies indicated that disease status in remission at transplantation and post transplantation could be prognostic factor indexes for improving OS. The pooled analysis of 7 trials for patients at transplantation in complete remission (CR) [15, 26, 40, 47, 50–52] and 3 trials for patients post-transplantation in CR [21, 50, 51] showed that patients at transplantation (HR = 0.43, 95% CI 0.29–0.63) or post transplantation (HR = 0.36, 95% CI 0.17–0.76) in CR had higher OS than those in non-CR.

High cytogenetic risk has been previously reported to be negative factor for OS and PFS. MM patients with high cytogenetic risk resisted to conventional chemotherapy, relapsed repeatedly after autologous stem cell transplantation (auto-SCT) and had a grim prognosis [53, 54]. In order to solve this problem, many new treatment strategies have been developed over the past few decades, including novel agents (bortezomib and lenalidomide) and double auto-SCT [55, 56]. Although overall response rates and OS have been increased, most patients still relapsed soon. All 14 trials involved in our study showed that high cytogenetic risk patients after allo-SCT had similar OS and PFS to those with standard-risk (RR = 0.83, 95% CI 0.67–1.03) [12, 14, 15, 21, 25, 31, 39, 42–44, 47, 50, 51, 57, 58]. However, most trials described the situation using descriptive language, only 5 provided concrete data of comparisons of OS between high cytogenetic risk patients and standard-risk patients [15, 25, 42, 44, 57]. A meta-analysis of OS provided by these 5 trials showed no statistical difference between high cytogenetic risk patients and standard-risk patients. No substantial heterogeneity was found among the included studies (p = 0.599, I^2^ = 0). These findings indicate allo-SCT overcomes the adverse prognosis of high cytogenetic risk.

EBMT centers reported that PBSCs have replaced bone marrow (BM) and then become the primary source of grafts since 2000 [8]. In every trial of the present meta-analysis, most allo-SCT were performed with PBSC as source of graft, while a few allo-SCT with BM. Only 4 studies provided the comparisons of OS between PBSC and BM, and the results indicated that use of PBSC had faster engraftment kinetics and quicker immune reconstitution than BM, but these advantages didn’t translate into higher OS (HR = 1.02, 95% CI 0.53–1.96) [31, 50, 59, 60].

Ten studies proved patients’ age was an independent predictor for shorter OS (HR = 1.03, 95% CI 1.01 1.05) [15, 20, 26, 31, 39, 44, 50, 52, 57, 61]. There was also evidence that patients receiving allo-SCT as first line treatment had better OS than patients receiving allo-SCT as salvage therapy (RR = 1.42, 95% CI 1.14–1.78) [33, 38, 58, 62].

### PFS

A total of 41 trials reported PFS with 1–217.2 months follow-up period [10, 11, 13–17, 19–33, 35–40, 42–49, 62–66]. The pooled estimates (95% CI) for PFS at 1, 2, 3 and 5 years were 51 (95% CI 38–64%), 40 (95% CI 32–48%), 34 (95% CI 27–41%), and 27 (95% CI 23–31%), respectively. High heterogeneity was found in these studies (p = 0, I^2^ = 92.4, 80.6, 94.5, 84.4%, respectively). Five studies [16, 19, 42, 46, 49] showed a trend that auto-allo-SCT had higher PFS than tandem-auto-SCT. But the pooled estimates from 5 trials showed that patients receiving auto-allo-SCT had the same PFS with those receiving tandem -auto-SCT (RR = 1.27, 95% CI 0.84–1.93). Three studies demonstrated patients receiving previous auto-SCT before allo-SCT transplanted had a significant advantage over those receiving allo-SCT directly (RR = 1.46, 95% CI 1.19–1.80) [17, 26, 65].

Subgroup analysis of 7 trials [26, 40, 47, 50–52, 60] for patients at transplantation in CR and 5 trials [21, 33, 50, 51, 60] for patients post transplantation in CR proved that patients at transplantation (HR = 0.59, 95% CI 0.44–0.78) or post transplantation (HR = 0.30, 95% CI 0.23–0.39) in CR had higher PFS, whereas there was no obviously different PFS between patients with RIC/NMA regimens and those with MA regimens [10, 13, 25, 29].

No evidence was found that genetic risk stratification (RR = 0.89, 95% CI 0.66–1.20) [15, 25, 42, 43, 57] and PBSC as source of graft (HR = 0.80, 95% CI 0.45–1.42) [31, 50, 60] would affect PFS. But there was a trend for worse PFS in the older patients arm (HR = 1.04, 95% CI 1.01–1.08) [26, 31, 39, 44, 50, 52, 57, 61] and those receiving allo-SCT as salvage therapy (RR = 0.36, 95% CI 0.25–0.51) [33, 38, 58, 62] compared with younger patients or those receiving allo-SCT as first line treatment.

### GVHD

In the trials involved in our meta-analysis, 55 [10–15, 17, 19–25, 27–36, 38–52, 57–65, 67–73] trials reported grades 2–4 acute GVHD (aGVHD) with the incidence varying from 2.3 to 69.6%, 38 trials [10, 11, 13, 14, 21, 23, 25, 27–30, 32–34, 36, 38, 40–42, 44, 45, 47–50, 52, 58, 59, 61, 63–65, 67–70, 72, 74] reported extensive chronic GVHD (cGVHD) with the incidence ranging from 5.3 to 79.3%, and 30 trials [11, 13, 22, 23, 25, 27–34, 36–38, 40, 42, 44, 45, 48–50, 58, 59, 63, 64, 68, 69, 72] reported limited cGVHD with the incidence varying from 5.1 to 46.3%. There were significant heterogeneity in the pooled estimates of the incidence of aGVHD (p = 0, I^2^ = 89.7%), extensive cGVHD (p = 0, I^2^ = 93.7%), as well as limited cGVHD (p = 0, I^2^ = 82.3%). The pooled estimates of aGVHD (grade 2/4) was 34% (95% CI 30–37%), extensive cGVHD was 36% (95% CI 31–42%), as well as limited cGVHD 20% (95% CI 16–23%). Many studies indicated aGVHD and cGVHD were two prognostic markers. To make conclusion more accurate, we pooled the HR provided in the multivariate regression, only referring to acute GvHD grades II–IV [40, 47, 60] and cGVHD [25, 36, 40, 51, 60, 62]. The pooled analyses showed that aGVHD was associated with shorter OS (HR = 2.25, 95% CI 1.55–3.27) compared with non-aGVHD, and cGVHD had better OS (HR = 0.32, 95% CI 0.19-–0.55) and PFS compared with non-cGVHD (HR = 0.45, 95% CI 0.29–0.69).

### TRM

Most studies involved in our analysis reported the results of TRM [10, 12, 13, 15–17, 19, 20, 22–34, 36–44, 47–49, 51, 57–60, 62, 63, 65, 66, 68, 71, 73, 74]. The pooled estimates (95% CI) for TRM at 100 days, 1, 2, 3 and 5 years were 12 (95% CI 5–18%), 18 (95% CI 14–21%), 21 (95% CI 17–25%), 20 (95% CI 13–26%), and 27 (95% CI 21–33%), respectively. Significant heterogeneity were founded (p = 0, I^2^ = 96.1%, 86.1, 68.8, 93.4, 90.5%, respectively). A large retrospective study indicated patients in CR at transplantation had lower risk of TRM than those in non-CR on multivariate analysis (HR 0.17, 95% CI 0.04–0.77) [15]. Subgroup analysis demonstrated a markedly reduced incidence of TRM in patients underwent planned auto-SCT before allo-SCT compared with those direct to allo-SCT [26, 40]. In contrast, there was increasing the risk of TRM in patients receiving auto-allo-SCT [16, 42, 49] or MA regimens [10, 13, 29, 45] compared with those receiving tandem-auto-SCT or RIC/NMA-HCT regimens. Since major transplant innovation and new technologies emerged in 2000, we compared the TRM of transplantation period 1990s and 2000s. The pooled estimates (95% CI) for 2000s’ TRM at 100 days, 1, 2, 3 and 5 years were 9 (95% CI 2–16%), 16 (95% CI 12–20%), 16 (95% CI 11–21%), 14 (95% CI 11–17%), and 22 (95% CI 11–32%), respectively. 1990s TRM at the same time were 12 (95% CI 4–21%), 19 (95% CI 13–25%), 23 (95% CI 18–28%), 24 (95% CI 14–33%), and 30 (95% CI 22–38%), respectively. Therefore, the risks of TRM from transplantation period 2000 s’ at 100 days, 1, 2, 3 and 5 years were all statistically lower than those from 1990 s’.

### RR, death rate and death causes

37 [10–13, 15, 19, 20, 22, 24, 26–28, 31–33, 36, 38, 40, 42–45, 47–50, 57–60, 64–67, 69, 72, 73] out of 61 trials reported the results of RR with the incidence ranging from 14.3 to 91.3%. The pooled estimate (95% CI) for RR was 50 (95% CI 45–55%). Significant heterogeneity was detected (p = 0, I^2^ = 91.3%). 3 trials showed patients after MA-SCT had lower RR than RIC/NMA-SCT [13, 25, 45], and 2 trials indicated a trend that RR was significantly lower in auto-allo-SCT patients compared with tandem-auto-SCT [42, 49]. Most trials reported death rate and the death cause [10–12, 14, 19, 20, 22, 24, 26, 28–33, 35, 36, 38, 40, 45, 47, 48, 51, 58–61, 63–65, 67, 69–71, 74]. The death rate ranged from 7.9 to 92.4%, and the pooled death rate was 51 (95% CI 45–57%). Substantial heterogeneity was found (p = 0, I^2^ = 96.7%). Disease progression was the major cause of the death (48%), followed by TRM (44%).

### Subgroup analyses and sensitivity analyses

Since major transplant innovation and new technologies happened in the year 2000, we took the year 2000 as a cut-off point of the subgroup analysis. We took the first year of transplantation period as a standard of 1990s and 2000s cut-off point, and found that substantial heterogeneity of 100-day TRM, 1-year TRM, 2-year TRM, 3-year TRM, and 3-year PFS mainly came from 1990s transplantation period (I^2^ = 97.5%, p = 0 for 1990s, I^2^ = 26.2%, p = 0.255 for 2000s; I^2^ = 93.6%, p = 0 for 1990s, I^2^ = 56.4%, p = 0.0031 for 2000s; I^2^ = 70.1%, p = 0.005 for 1990s, I^2^ = 13.2%, p = 0.327 for 2000s; I^2^ = 96.6%, p = 0 for 1990s, I^2^ = 0%, p = 0.552 for 2000s; I^2^ = 97.4%, p = 0 for 1990s, I^2^ = 62%, p = 0.007 for 2000s; respectively). This phenomenon most probably due to 1990s subgroup including a few patients performed allo-SCT after the year 2000. The articles involved didn’t provide the individual data of those patients, so we failed to separate out these small amount of patient from 1990s subgroup.

Sensitivity analyses were performed by excluding a study at a time in turn, and pooling the outcomes of the remaining studies. No material changes happened in all results.

### Publication bias

We failed to identify obvious asymmetry with the exception of 5-year PFS, 5-year OS and exGVHD in all the funnel plots through visual inspection. In line with the funnel plots, Egger test find substantial publication bias’ evidence in 5-year PFS, 5-year OS and exGVHD (p = 0.046, p = 0.036, p = 0.013, respectively).

## Discussion

Since the first allo-SCT was performed by Donnall Thomas in 1957 [75], it has been chosen to be a salvage regimen for relapsed or refractory MM patients for several decades [76, 77]. However, a large number of articles reported that allo-SCT failed to be used widely because of unusable donors, high TRM and GVHD. Compared with auto-SCT, allo-SCT is still desirable due to the higher rates of molecular responses, longer-term disease control and graft-versus-myeloma effect despite higher TRM. Additionally, the increasing use of RIC/NMA regimens and PBSC as source of graft, advances in supportive care and effective infection prevention programs since 2000 may facilitate allo-SCT to be a potential way to cure MM. Therefore, we performed a systemic review and meta-analysis of 61 clinical trials reported between 2007.01.01 and 2017.05.03 involving 8698 adult patients to examine the efficacy and safety of allo-SCT for MM.

High-risk (HR) patients seem to be relatively resistant to novel agents, and had only short-term or no response to high-dose chemotherapy with or without following auto-SCT [55, 78–80]. In addition, some recent studies also reported high-risk patients may acquire new clonal abnormalities, showing rapidly progressing relapses after induction therapy followed by upfront ASCT, even evolving into extramedullary relapses or secondary plasma cell leukemia [81, 82]. The International Myeloma Working Group (IMWG) has demonstrated that newly diagnosed high-risk patients treated with conventional therapies had a median overall survival (OS) of only 2- and 4-year OS was only 33% [83]. Medical Research Council (MRC) Myeloma IX trial showed ultra-high risk MM defined by ISS II or III in the presence of > 1 adverse lesion, including adverse IGH translocations, + 1q21 and del(17p13), have a particularly poor outcome (a median PFS of only 9.9 months and a median OS of 19.4 months) after being treated with auto-SCT [84]. In contrast, all 14 trials in our study showed that patients with high cytogenetic risk after allo-SCT had similar OS and PFS compared to those with standard risk, suggesting that allo-SCT may overcome the adverse prognosis of high cytogenetic risk. Furthermore, a included prospective study showed that after performing auto-SCT followed by allo-SCT, high risk patients with del(17p)/t(4;14) had similar remission rate, PFS, OS and relapse rate to those without del(17p)/t(4;14). Even ultra-high risk patients obtained molecular complete remission [43]. Importantly, Barlogie et al. showed flat survival curves between 4 and 10 years post allo-SCT, which suggested a proportion of these high risk patients may experience prolonged disease control or perhaps cure [85].

Some negative factors were especially important consideration in the counseling, implementation, and post treatment management of allo-SCT. We found TRM at 100 days and 1 year were 12 (5–18)% and 18 (14–21)%, respectively. Although compared with 50% TRM when the allo-SCT was initially performed [8], the present TRM has been drastically reduced, it was still too high for standard-risk patients. It should be mentioned that the patients in our study were heavily pretreated, which probably explains the high TRM observed. Indeed, more than half of patients had received at least 2 prior lines of treatment and 25% had at least 2 prior auto-SCT. The costs of allogenic transplant for MM are greater than those for chemotherapy and autologous transplantation [86, 87]. The median number of hospital days for allo-SCT are longer than that for auto-SCT [88]. The article we included did not mention quality of life (QOL), but many other articles showed comparisons between patients after transplantation and adults without cancer. These studies showed that patients who underwent transplantation have low or moderate impairment in physical, social, psychological, and emotional functioning, as well as overall QOL [89–91]. The decline in overall QOL for auto-SCT was transient, but it was a longer term for allo-SCT [92]. However, Bush et al. reported that 80% of survivors had returned to work or school to resume their roles at home and in the community at 2 years after transplantation [93]. Specifically, patient reported benefits include an enhanced appreciation for life, different priorities, love and appreciation for family and friends, and greater religious or spiritual beliefs [90, 94, 95]. These data suggest that patients are often able to reinterpret the adversity of allo-SCT into a meaningful life narrative despite reduced QOL. Though 5-year PFS of 27% after allo-SCT isn’t an ideal outcome, it’s still better than 5-year PFS of only 19% after tandem auto-SCT reported by other articles [49]. High-risk patients—either upfront or relapsed—may be candidates for the allo-SCT treatment, when the risk of disease progression may outweigh the transplant-related risks. A large number of prospective randomized controlled trials were needed to prove the benefits of these therapeutic options.

**Fig. 1:**
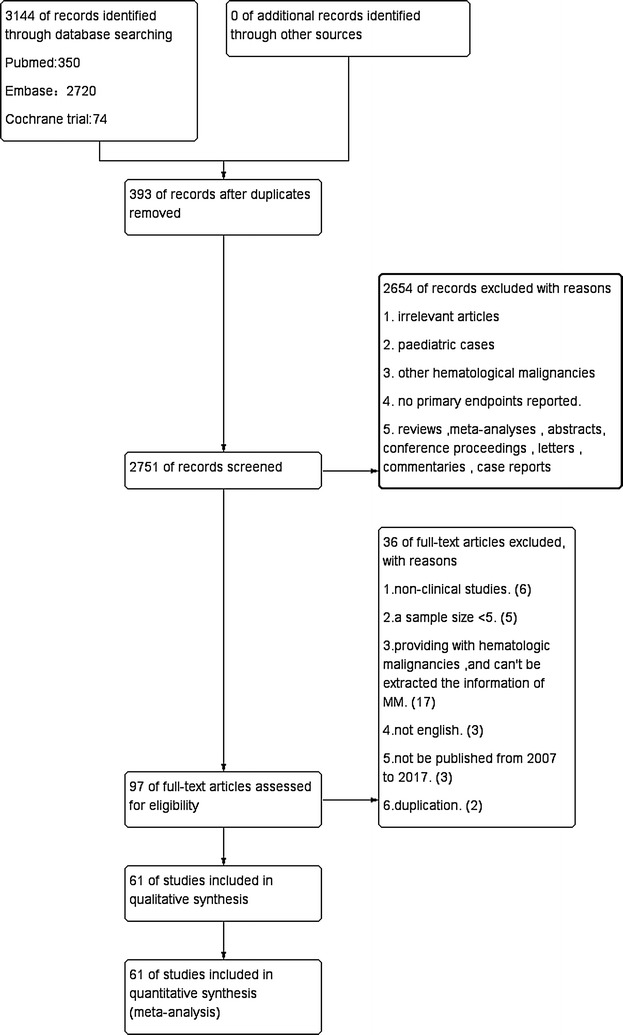
Flow diagram of study selection method

**Fig. 2:**
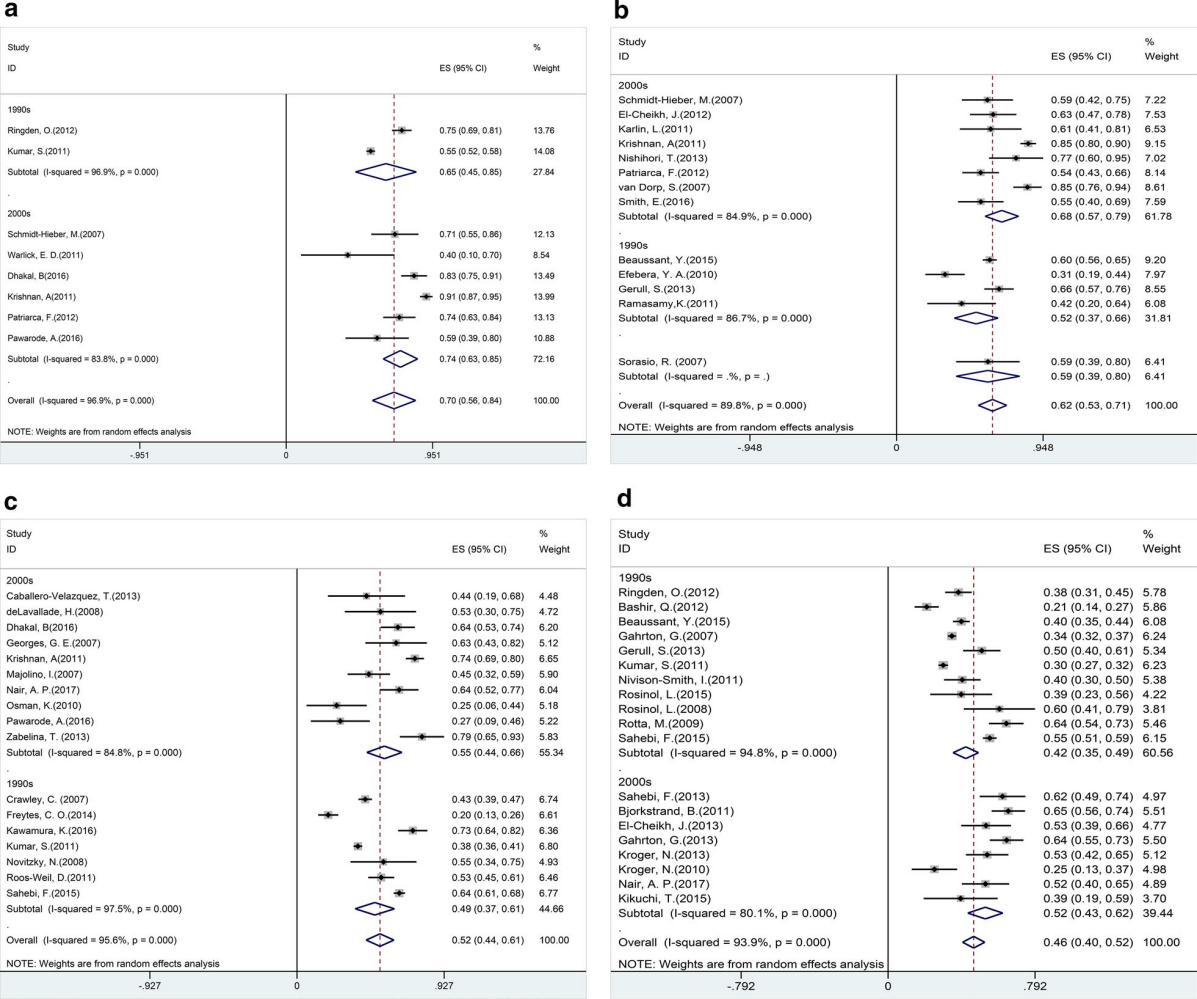
Individual and pooled weighted incidence of overall survival (OS) at 1 year (**a**), 2 years (**b**), 3 years (**c**), and 5 years (**d**), stratified by transplantation period. Effect size (ES) is odds ratio or relative risk depending on the study

**Fig. 3:**
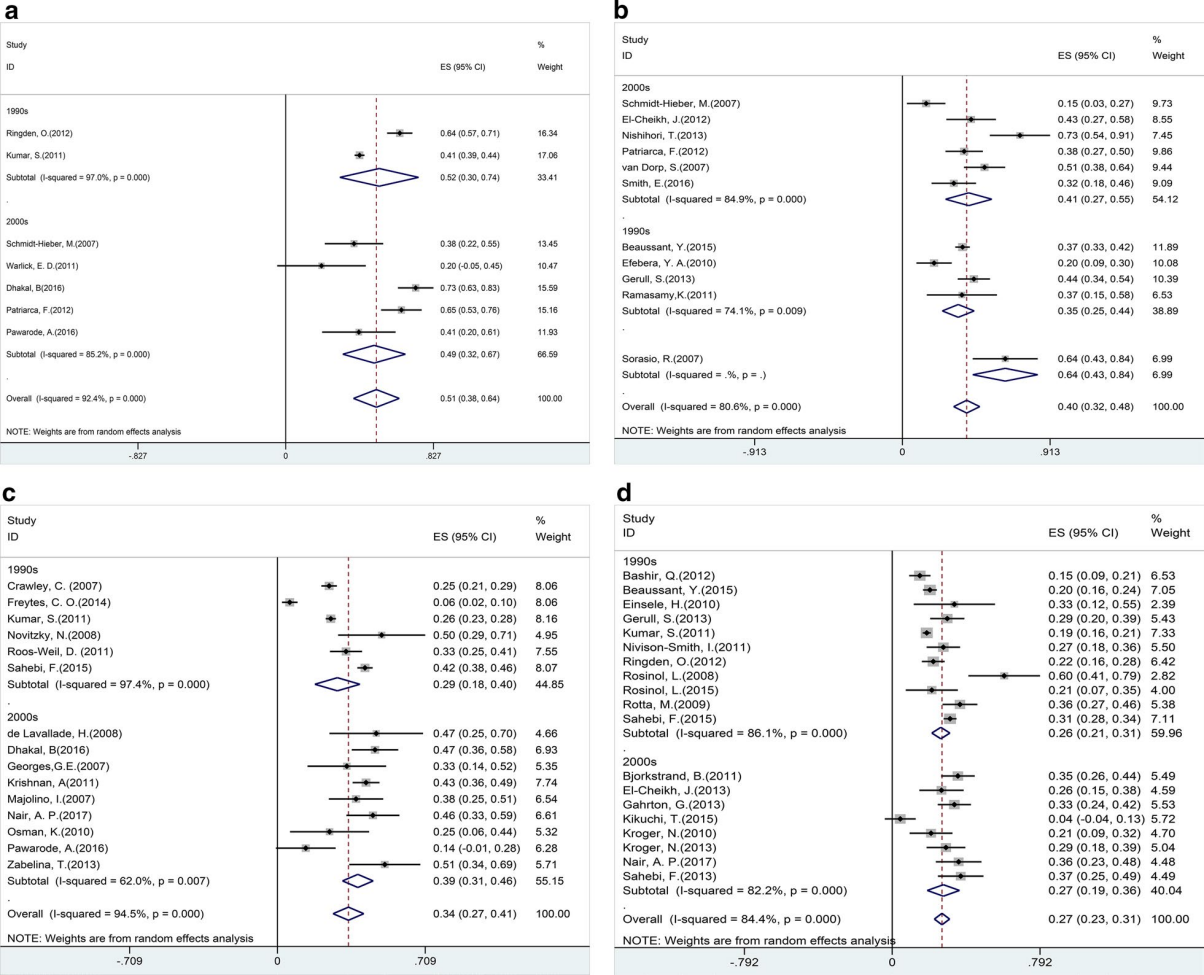
Individual and pooled weighted incidence of progression-free survival (PFS) at 1 year (**a**), 2 years (**b**), 3 years (**c**), and 5 years (**d**), stratified by transplantation period. Effect size (ES) is odds ratio or relative risk depending on the study

**Fig. 4:**
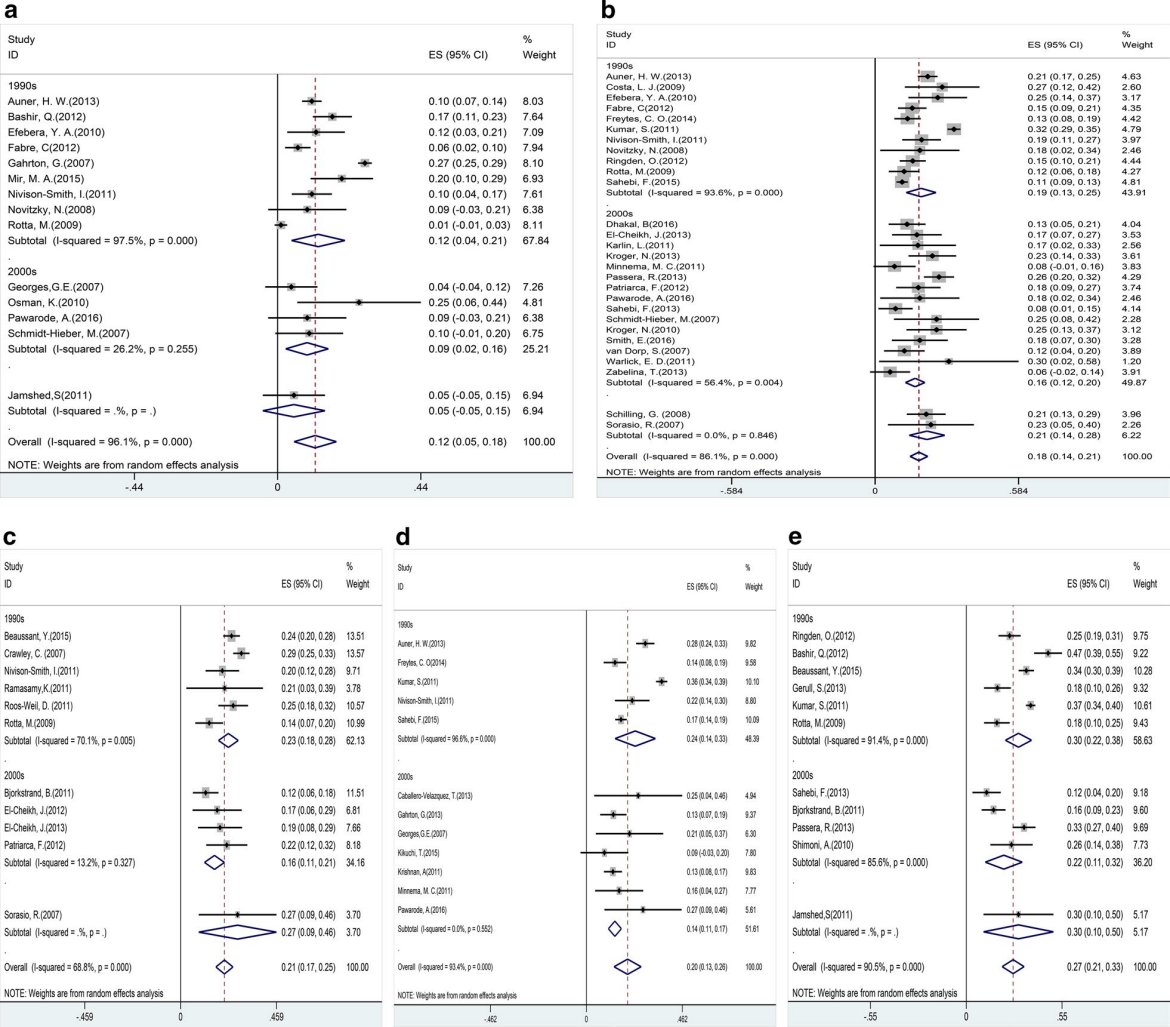
Individual and pooled weighted incidence of treatment-related mortality (TRM) at 100 days (**a**), 1 year (**b**), 2 years (**c**), 3 years (**d**), and 5 years (**e**), stratified by transplantation period. Effect size (ES) is odds ratio or relative risk depending on the study

**Fig. 5:**
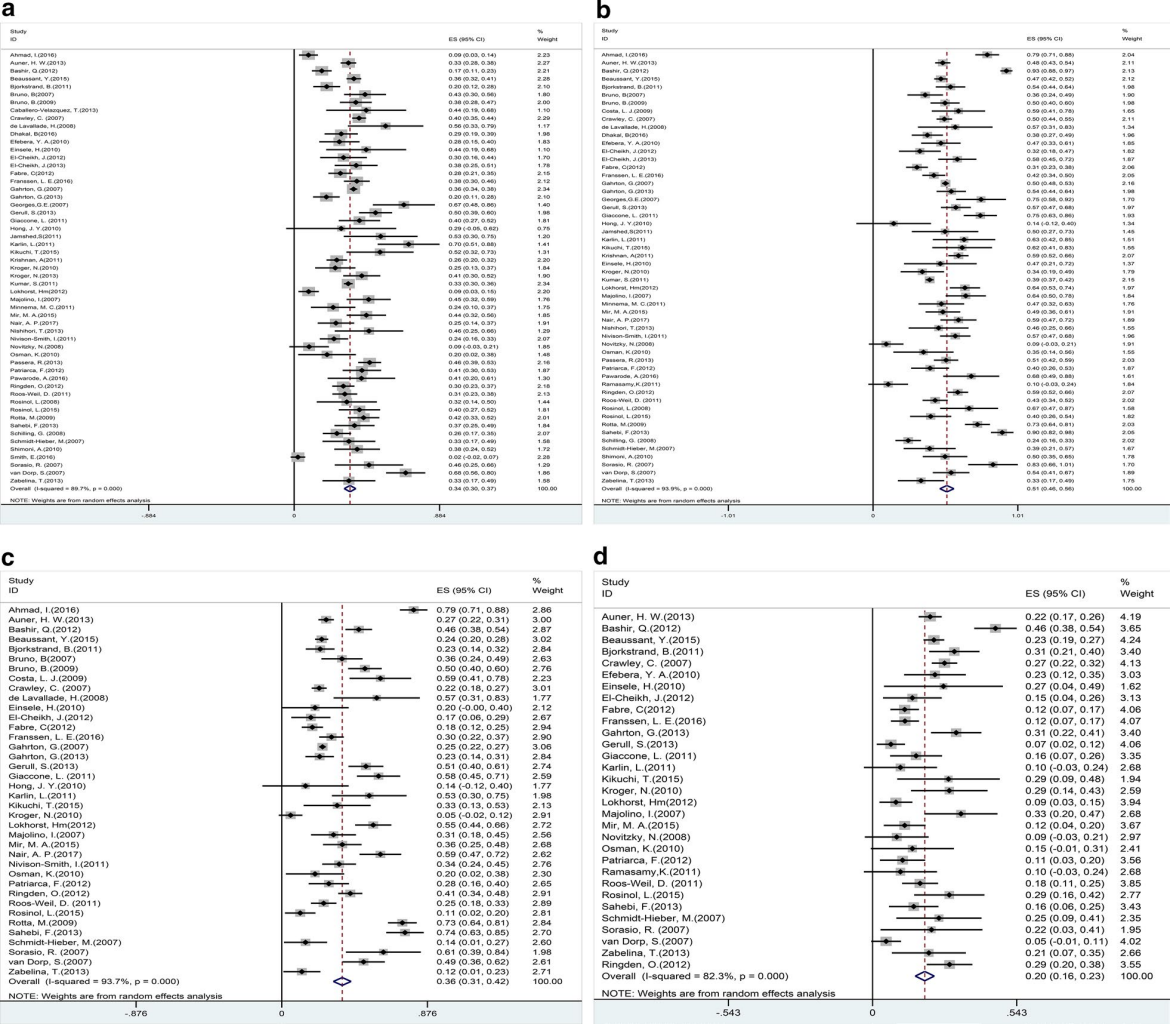
Forest plot of the risk of grades II–IV acute graft-versus-host disease (GVHD) (**a**), chronic GVHD (**b**), extensive cGVHD (**c**), limited cGVHD (**d**). Effect size (ES) is odds ratio or relative risk depending on the study

**Fig. 6:**
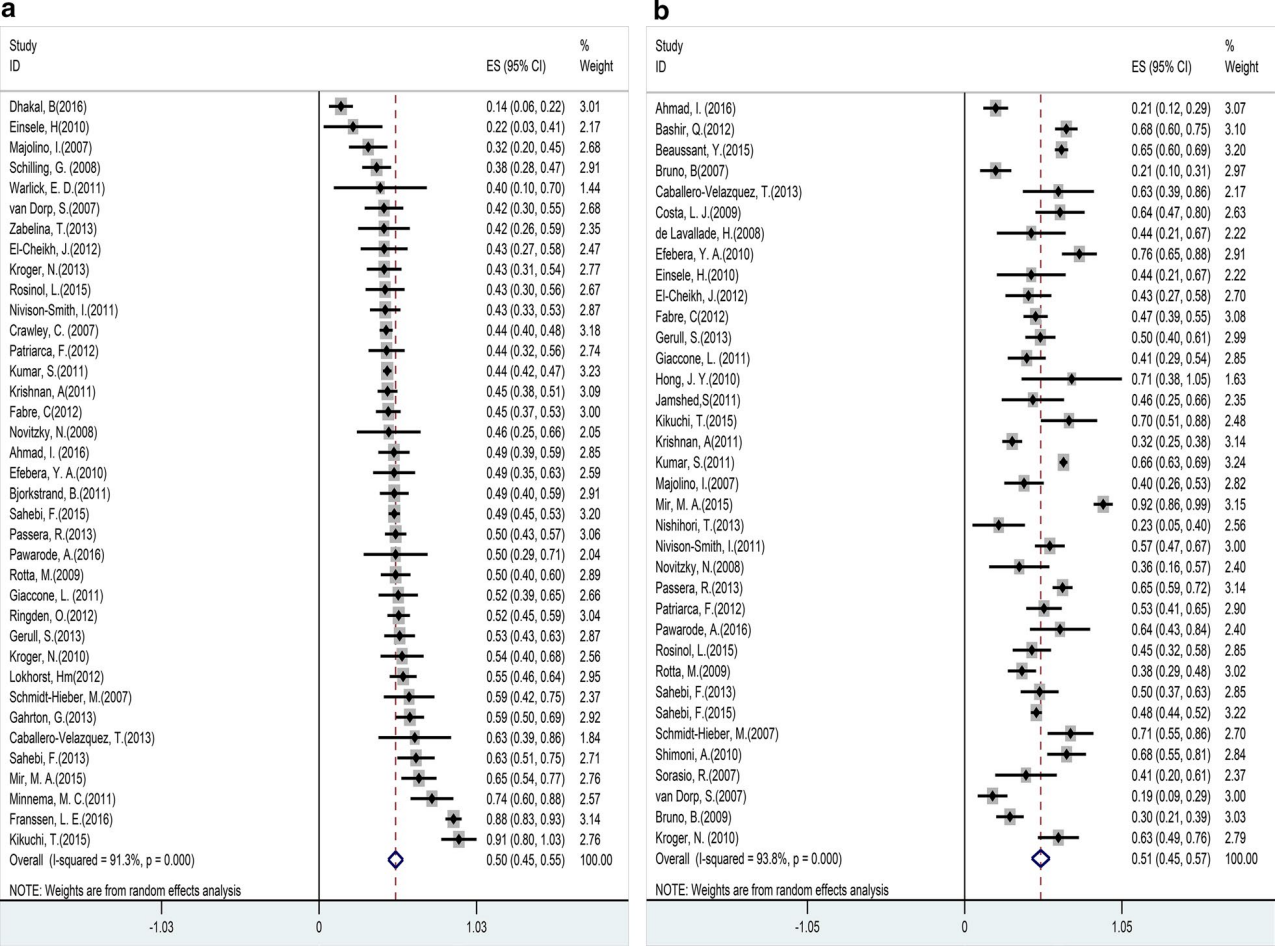
Forest plot of the risk of relapse (**a**) and death (**b**). Effect size (ES) is odds ratio or relative risk depending on the study

Even when limited to patients with high risk MM or as first or second line salvage, the risk of acute and chronic GVHD and the high rates of recurrence after allograft need to be addressed to make allografting a readily acceptable treatment options for MM patients (Figs. 1, 2, 3, 4, 5, 6).

We found aGVHD was an independent predictor for shorter OS and PFS, while cGVHD might prolong survival due to an accompanying GvM effect and can thus in a mild form be regarded as an advantageous feature. However, some studies have reported cGVHD was also related to chronic diarrhea, appetite loss and inferior QOL [90, 96, 97], and was the most important cause of TRM. Novel strategies for acute and chronic GVHD prevention need to be explored in the setting of allografting for MM. Kroger et al. reported High-dose ATG could decrease the risk of aGVHD without the improvement of relapses [64]. T cell depletion using CD34 selection also could reduce the occurrence of GVHD [98].

Currently, it is noticeable that allo-SCT still remains high relapse rate [50 (45–55)%] and disease progression dominates the cause of the death (48%). Post-transplant immune treatment may be the key to sustain remission, prolong PFS and reduce relapse rate。Post-transplant therapies consist of donor lymphocyte infusions (DLI), possibly combined with immune stimulatory drugs [99, 100]. Monoclonal antibodies, checkpoint inhibitors, vaccines and additional cellular therapies such as CAR-T-cells and NK-cell therapy could be used in the future [101]. They specifically target the remaining myeloma cells and limit the risk of augmenting GVHD [101]. Use of novel agents post-allografting has also been explored. For example, due to its dual effect—preventing GVHD and preserving GVM—bortezomib may be ideal to use upfront in the conditioning regimen and/or for consolidation and maintenance in the allogeneic transplant setting [102, 103].

We identified many prognostic factors that may affect the outcome of allo-SCT. Since RIC/NMA was firstly introduced in 2000, MA have been gradually replaced to reduce TRM. Although the widespread adoption of RIC/NMA regimens failed to bring about better OS and PFS in the present meta-analysis, but it evidently enhanced patients’ over QOL [104, 105]. PBSC serving as the source of hematopoietic stem cells increased donors’ availability and made allo-SCT more accessible. We found PBSC was associated with faster engraftment kinetics and quicker immune reconstitution, but less benefits in OS and PFS when compared with BM. In addition, three large retrospective studies [17, 30, 32] indicated unrelated donors in MM patients had similar engraftment rate to HLA-matching donors and acceptable TRM. Unrelated donors owing to a better donors’ availability may be the feasible option. In our analysis, four studies indicated that compared with direct allo-SCT, planned autologous transplantation before allo-SCT had lower TRM as well as higher OS and PFS. Since 2000, nearly all allo-SCTs have adopted RIC/NMA regimens, which had insufficient cytoreduction. To our opinion, planned auto-SCT leads to sufficient cytoreduction and offset the disadvantage of RIC/NMA, thereby having better anti-MM effect than direct allo-SCTs. We showed the quality of response was also an independent prognostic factor for better PFS and OS before and after allo-SCT. Future therapeutic strategies should pay more attention to patients in non-CR state after the induction therapy and post-transplantation therapy. Adoptive immunotherapy alone or in combination with novel drugs can be applied to help these patients reach CR state. We found patients’ age may be an independent predictor for shorter PFS and OS. A large prospective study reported donor’s age > 50 years mean worse OS (HR = 1.99, 95% CI 1.22–3.25), which emphasized increasing donor’s age impaired donor stem cells’ repopulation and homing abilities [58].

Several limitations should be considered in this meta-analysis. (1) Because the total sample size applied to perform a specific analysis was too small, we included the results of univariate analysis. Therefore, the conclusion was inaccurate in some way. (2) Significant heterogeneity was founded in the present study. We performed subgroup analyses and sensitivity analyses to explore the causes, and found the transplantations period of 1990s may be one source of significant heterogeneity. Because the 1990 s’ subgroup included a few patients who had allo-SCT after the year 2000, and the articles involved didn’t provide the individual data of those patients, we were incapable to separate the data out. In addition, the heterogeneity may come from different conditioning regimens, GVHD prophylaxis and patient selection bias (age, comorbidity, stage of disease) among different studies. But 95% confidence interval of all results was narrow, which meant the conclusion was credible in a way (Tables 1, 2).

**Table 1:** Subgroup analysis and the pooled HR for patients

Outcomes	No. of studies	Heterogeneity (I^2^), %	HR (95% CI)	p for heterogeneity
OS				
Post-transplantation in CR	3	63.3	0.36 (0.17, 0.76)	0.066
At transplantation in CR	7	47.6	0.43 (0.29, 0.63)	0.076
Over 50	10	61.4	1.03 (1.01, 1.05)	0.006
PBSC	4	66.7	1.02 (0.53, 1.96)	0.029
aGVHD	3	0	2.25 (1.55, 3.27)	0.814
cGVHD	4	39.4	0.32 (0.19, 0.55)	0.175
PFS				
Post-transplantation in CR	5	0	0.30 (0.23, 0.39)	0.609
At transplantation in CR	7	35.7	0.59 (0.44, 0.78)	0.156
Over 50	8	69.4	1.04 (1.01, 1.08)	0.002
PBSC	3	47.2	0.80 (0.45, 1.42)	0.150
aGVHD	2	0	1.27 (0.84, 1.94)	0.660
cGVHD	5	39.5	0.45 (0.29, 0.69)	0.158

**Table 2:** Subgroup analysis and the pooled RR for patients

Outcomes	No. of studies	Heterogeneity (I^2^), %	RR (95% CI)	p for heterogeneity
OS				
Auto-allo vs only-allo	2	0	1.28 (1.11, 1.49)	0.435
Auto-allo vs tandem auto	6	79	0.91 (0.77, 1.06)	0.000
MA vs RIC	4	38.8	1.14 (0.95, 1.36)	0.179
High-risk vs standard-risk	5	0	0.83 (0.67, 1.03)	0.599
First-line vs salvage therapy	4	42.6	1.42 (1.14, 1.78)	0.156
PFS				
Auto-allo vs only-allo	3	17.6	1.46 (1.19, 1.80)	0.297
Auto-allo vs tandem auto	5	79.9	1.27 (0.84, 1.93)	0.001
MA vs RIC	4	61.9	1.32 (0.95, 1.83)	0.049
High-risk vs standard-risk	5	6.1	0.89 (0.66, 1.20)	0.372
First-line vs salvage therapy	4	0	2.80 (1.97, 3.97)	0.948
TRM				
Auto-allo vs only-allo	2	0	0.41 (0.27, 0.61)	0.420
Auto-allo vs tandem auto	3	0	6.09 (2.92, 12.7)	0.999
MA vs RIC	4	26.0	1.48 (1.14, 1.92)	0.256
RR				
Auto-allo vs only-allo	3	75.9	0.80 (0.51, 1.24)	0.016
Auto-allo vs tandem auto	2	48.1	0.63 (0.50, 0.78)	0.165
MA vs RIC	3	65.6	0.64 (0.43, 0.95)	0.055

Importantly, this study had its own remarkable profits. (1) To our knowledge, the present meta-analysis was the first and largest comprehensive review of the role of allo-SCT in the treatment of MM patients. We found the most suitable subgroup of patients for allo-SCT and the best therapeutic time window of allo-SCT in MM patients. (2) In our analysis, 29/61 studies scored 5 that was deemed as good quality, resulting in a more preferable conclusion to some extent. (3) We investigated many factors which may emerge as predictors of survival outcomes in MM patients after allo-SCT. The present meta-analysis may provide indications to policy makers and holistic clinicians in applying allo-SCT to clinical practice (Additional files 1, 2, 3, 4, 5, 6, 7, 8, 9, 10, 11, 12, 13, 14, 15, 16, 17).

## Conclusion

Due to the lack of consistent survival benefit, allo-SCT should not be considered as a standard of care for newly diagnosed and relapsed standard-risk MM patients. However, for patients with high-risk MM who have a poor long-term prognosis, allo-SCT may be a strong consideration in their initial course of therapy or in first relapse after chemotherapy, when the risk of disease progression may outweigh the transplant-related risks. A large number of prospective randomized controlled trials were needed to prove the benefits of these therapeutic options.

## Additional files

**Additional file 1: Table S1.** Characteristics of studies included in the meta-analysis.

**Additional file 2: Table S2.** Patients’ transplant outcomes of individual clinical trials.

**Additional file 3: Table S3.** Quality assessment of individual clinical trials.

**Additional file 4: Table S4.** Begg and Egger test of studies included in the meta-analysis.

**Additional file 5: Figure S1.** Forest plot of the subgroup overall survival (OS) benefit (auto-allo vs only-allo, auto-allo vs tandem auto, MA vs RIC, high-risk vs standard-risk, first-line vs salvage therapy) (a), (post-transplantation in CR vs in non-CR, at transplantation in CR vs in non-CR, over 50 vs under 50, PBSC vs BM, aGVHD vs non-aGVHD, cGVHD vs non-cGVHD) (b).

**Additional file 6: Figure S2.** Forest plot of the subgroup progression-free survival (PFS) benefit (auto-allo vs only-allo, auto-allo vs tandem auto, MA vs RIC, high-risk vs standard-risk, first-line vs salvage therapy) (a), (post-transplantation in CR vs in non-CR, at transplantation in CR vs in non-CR, over 50 vs under 50, PBSC vs BM, aGVHD vs non-aGVHD, cGVHD vs non-cGVHD) (b).

**Additional file 7: Figure S3.** Forest plot of the subgroup relapse rate (RR) (a) and death rate benefit (b) (auto-allo vs only-allo, auto-allo vs tandem auto, MA vs RIC).

**Additional file 8: Figure S4.** Funnel plot of overall survival (OS) at 1 year (a), 2 years (b), 3 years (c), and 5 years (d).

**Additional file 9: Figure S5.** Funnel plot of progression-free survival (PFS) at 1 year (a), 2 years (b), 3 years (c),and 5 years (d).

**Additional file 10: Figure S6.** Funnel plot of treatment-related mortality (TRM) at 100 days (a), 1 year (b), 2 years (c), 3 years (d), and 5 years (e).

**Additional file 11: Figure S7.** Funnel plot of grade II–IV acute graft-versus-host disease (GVHD) (a), chronic GVHD (b), extensive cGVHD (c), limited cGVHD (d).

**Additional file 12: Figure S8.**Funnel plot of relapse rate (a) and death (b).

**Additional file 13: Figure S9.** Sensitivity analysis diagram of overall survival (OS) at 1 year (a), 2 years (b), 3 years (c),and 5 years (d).

**Additional file 14: Figure S10.** Sensitivity analysis diagram of progression-free survival (PFS) at 1 year (a), 2 years (b), 3 years (c),and 5 years (d).

**Additional file 15: Figure S11.**Sensitivity analysis diagram of treatment-related mortality (TRM) at 100 days (a), 1 year (b), 2 years (c), 3 years (d),and 5 years (e).

**Additional file 16: Figure S12.** Sensitivity analysis diagram of grade II–IV acute graft-versus-host disease (GVHD) (a), chronic GVHD (b), extensive cGVHD (c), limited cGVHD (d).

**Additional file 17: Figure S13.** Sensitivity analysis diagram of relapse rate (a) and death (b).

## Abbreviations

MM: multiple myeloma
OS: overall survival
PFS: progression-free survival
TRM: treatment-related mortality
GVHD: graft-versus-host disease
aGVHD: acute GVHD
cGVHD: chronic GVHD
exGVHD: extensive cGVHD
limGVHD: limited cGVHD
RR: relapse rate
Allo-SCT: allogeneic stem cell transplantation
Auto-SCT: autologous stem cell transplantation
Auto-allo: planned autologous transplantation before allogeneic stem cell transplantation
Only-allo: direct allogeneic stem cell transplantation
Tandem auto: tandem autologous stem cell transplantation
MA: myeloablative conditioning
RIC: reduced-intensity conditioning
CR: complete remission
PBSC: peripheral blood stem cell
BM: bone marrow
GVM: graft-versus-myeloma
EBMT: European Group for Blood and Marrow Transplantation
IMiDs: immune-modulatory drugs
