# Supplementary material for: Allogeneic stem-cell transplantation for multiple myeloma: a systematic review and meta-analysis from 2007 to 2017
Source: Cancer Cell Int. 2018 Apr 23;18:62. doi: 10.1186/s12935-018-0553-8 (PMC5913895; doi:10.1186/s12935-018-0553-8)
Supplement: Supplementary file 1 — Additional file 1: Table S1. Characteristics of studies included in the meta-analysis. [file 12935_2018_553_MOESM1_ESM.docx]

| Author(years) | N | Gender  (male/female) | Median age(range), years | Median follow-up(range),months | retrospectie | conditioning regimens | strategies for GVHD prophylaxis | Source of the transplanted stem cell(peripheral blood stem cell/bone marrow) |
| --- | --- | --- | --- | --- | --- | --- | --- | --- |
| Majolino, I.  (2007)^1^ | 53 | 35/18 | 52 (38 – 68) | 22(5-47) | + | TT+Flu+MEL | CsA+ MTX | PBSC44  BM9 |
| Novitzky, N.  (2008)^2^ | 22 | 10/12 | 45(37-56) | 1101days(385-5309days) | _ | TBI+Mel  Bu+Mel+CY | T-cell depletion +CAMPATH-1 antibody | PBSC15  BM7 |
| Ringden, O.  (2012)^3^ | 177 | 102/75 | 51 (24–69) | 29 (3–98) | + | NAM: TBI/Fludarabine+TBI  RIC: TBI/Mel/BU/Cy | CsA ±MTX 52  FK506±MTX 23  CSA+MMF 86  FK506+MMF 2  Campath±other 2  Other/unknown12 | PBSC173  BM4 |
| Sahebi, F.  (2013)^4^ | 60 | 40/20 | 51(32.3- 66.1) | 9.8(3.8–12 ) | _ | flu-mel/TBI | NA | PBSC58  BM2 |
| Schmidt-Hieber, M.(2007)^5^ | 34 | 23/11 | 51.5(33–65) | 708days(range 60–1729  days) | + | flu/treosulfan | CSA:8  CSA+MTX :15  CSA+MMF:9  CSA+MTX+MMF:2 | PBSC30  BM4 |

| Author(years) | Previous auto-SCT | Regimen(MA/RIC) | Status at  transplantations | Cytogenetic risk | Transplantation period | Donor type | Stage ISS | Score |
| --- | --- | --- | --- | --- | --- | --- | --- | --- |
| Majolino, I.  (2007)^1^ | 33 | 100% RIC | NA | NA | 2001-2005 | A fully-  matched HLA | NA | 4 |
| Novitzky, N.  (2008)^2^ | 3 | 100%MA | NA | NA | 1990-2006 | HLA-identical siblings | NA | 4 |
| Ringden, O  (2012)^3^ | NA | NMA:98  RIC :79 | CR/PR 127  MR10  SD16  PD 2  Missing 22 | NA | 1997-2005 | HLA-identical sibling | NA | 5 |
| Sahebi, F.  (2013)^4^ | 39 | NA | CR11  PR27  SD8  PD14 | Standard-risk 49  High-risk4  Unknown 7 | 2000-2008 | Sibling donor 55  MUD 5 | NA | 4 |
| Schmidt-Hieber, M.(2007)^5^ | 31 | NA | CR3  PR15  MR1  SD2  PD12  Plateau1 | NA | 2001-2005 | MRD 16  MUD 17  MMUD 1 | NA | 5 |

| Author(years) | N | Gender  (male/female) | Median age(range), years | Median follow-up(range),months | retrospective | conditioning regimens | strategies for GVHD prophylaxis | Source of the transplanted stem cell(peripheral blood stem cell/bone marrow) |
| --- | --- | --- | --- | --- | --- | --- | --- | --- |
| Ahmad, I.  （2016）^6^ | 92 | 52/40 | 52(39-64) | 8.8 years | _ | Flu+CY | tacrolimus + MMF | NA |
| Auner, H. W. (2013)^7^ | 413 | 265/148 | 54.1 (26.9–69.6) | NA | + | Flu+ TBI 122  Flu+ Mel 88  Flu+ BU 75  Flu+ CY 38  Others 90 | CsA 365  MMF 131  MTX 101 | PBSC373  BM 35  PBSC + BM 1  Cord blood 4 |
| Bashir, Q.  (2012)^8^ | 149 | 87/62 | 50 (28–70) | 28.5 (3–164) | + | MAC:  Flu+Mel7  Mel6 /Mel+TBI 10  Bu+CSA+TT 6  Others 9  RIC:  Flu+Mel 99  TBI 6  Others 5 | Cy 4  Cy+MTX 12  Cy+MMF 6  None6  Tacrolimus 6  Tacrolimus-MTX 104 | PBSC 100  BM 46  Cord blood 3 |
| Beaussant, Y.(2015)^9^ | RIC:397  MAC:49 | RIC:243/154  MAC:36/13 | RIC: 54.6 (27.4-69.6)  MAC: 47.1 (25.7-63.0) | 33.6 (0 to 164.5) | + | MAC:  CY+TBI:22  BU+CY:9/CY+FLU:6  MEL+TBI:4/BU+FLU:2  RIC:  BU+FLU199/FLU+TBI:114  FLU+MEL:32/TBI:22 | CSA  CSA-MMF  CSA-MTX | RIC:  BM39  PBSC 358  MAC:  BM 19  PBSC 30 |
| Bjorkstrand, B.(2011)^10^ | 108 | 65/43 | 54(34-66) | 61(21-91) | + | TBI+FLU | CSA-MMF | NA |
| Bruno, B  (2007)^11^ | 58 | 30/28 | 55(34-65) | 45(21-90) | _ | TBI | NA | 100%PBSC |

| Author(years) | Previous auto-SCT | Regimen(MA/RIC) | Status at  transplantations | Cytogenetic risk | Transplantation period | Donor type | Stage ISS | Score |
| --- | --- | --- | --- | --- | --- | --- | --- | --- |
| Ahmad, I.  （2016）^6^ | 92 | 100%NMA | ⩾ VGPR 52  PR 27  <PR 11  Not available 2 | NA | 2001-2010 | 6/6HLA-matched sibling donor | I 17  II 5  III 6  Unknown 64 | 4 |
| Auner, H. W.  (2013)^7^ | 413 | 100%RIC | >PR 42  PR 221  <PR 124 | NA | 1999-2008 | Sibling 237  Other relative 15  UD 157 | NA | 5 |
| Bashir, Q  (2012)^8^ | 91 | RIC110  MAC38 | CR3 /VGPR17  PR 56/SD29  PD43 | Poor-risk36 | 1985-2010 | RD114  UD35 | NA | 5 |
| Beaussant, Y.  (2015)^9^ | RIC:373  MAC:39 | RIC:397  MAC:49 | RIC:  CR 62/PR 276  SD27/PD32  MAC: CR 11 / PR 32  SD 2 / PD 4 | NA | 1999-2009 | RIC:Identical sibling 298  MUD 99  MAC:Identical sibling 33  MUD 16 | NA | 5 |
| Bjorkstrand, B.(2011)^10^ | 108 | 100%RIC | CR:7  PR:83  SD:18 | NA | 2001-2005 | HLA-identical sibling donor | NA | 4 |
| Bruno, B  (2007)^11^ | 58 | 100%NMA | CR:8  PR:36 | NA | 1998-2004 | HLA-identical sibling | NA | 4 |

| Author(years) | N | Gender  (male/female) | Median age(range), years | Median follow-up(range),months | retrospective | conditioning regimens | strategies for GVHD prophylaxis | Source of the transplanted stem cell(peripheral blood stem cell/bone marrow) |
| --- | --- | --- | --- | --- | --- | --- | --- | --- |
| Sorasio, R. (2007)^12^ | 22 | 11/11 | 54 (38–63) | 20 (10–30) | _ | FLU+TBI | CSA+MMF | 100%PBSC |
| Crawley, C.  （2007）^13^ | RIC320  MAC196 | RIC;210/110  MAC:123/73 | RIC:51 (31-66)  MAC:45 (29-59) | NA | + | RIC:MEL+BU+CY+TBI  MAC:MEL+TBI  CY+TBI | CSA  CSA + MTX  CSA+TCD CSA/MTX TCD | RIC:BM 55/ PBSC 265  MAC: BM 80 /PBSC 116 |
| Hong, J. Y.  (2010)^14^ | 7 | 3/4 | 40(32-46) | 376days | + | FLU+MEL | CSA+MTX | NA |
| Warlick, E. D.(2011)^15^ | 10 | NA | NA | 30.6 (3.3-81) | + | FLU+CY+TBI+ATG | CSA+MMF | NA |
| Jamshed,S (2011)^16^ | 22 | 14/8 | 53(36-65) | 44.6(1-69) | + | FLU+CY  CY+doxorubicin +etoposide | CSA+MTX  CSA | NA |
| Einsele, H (2010)^17^ | 18 | 14/4 | 44(29-53) | 105(23-145) | _ | TBI+BU+CY | ATG  CSA+MTX | PBSC5  BM13 |
| Bruno, B.  (2009)^18^ | 96 | 52/44 | 54 (30-65) | 5 years ( 0.7-8.4) | _ | TBI+MMF+CY | NA | 100%PBSC |
| Caballero-Velazquez, T.(2013)^19^ | 16 | 14/2 | 51(37-64) | 837 days | _ | Bz+MEL+FLU | CSA+MTX | 100%PBSC |

| Author(years) | Previous auto-SCT | Regimen(MA/RIC) | Status at  transplantations | Cytogenetic risk | Transplantation period | Donor type | Stage ISS | Score |
| --- | --- | --- | --- | --- | --- | --- | --- | --- |
| Sorasio, R. (2007)^12^ | 21 | 100%NMA | CR 2  PR 13  Refractory disease 7 | NA | NA | Donors were matched for HLA-A, B, C, DRB1 and DQB1 by high-resolution typing | NA | 5 |
| Crawley, C.  （2007）^13^ | RIC245  MAC21 | RIC320  MAC196 | RIC: CR 41  PR 189  SD/PD 90  MAC: CR 29  PR 125  SD/PD 42 | NA | 1998-2002 | RIC:Sibling 262  MRD4  MMRD 5  UD 49  MAC: Sibling 168  MRD 8  MMRD 5  UD 15 | NA | 5 |
| Hong, J. Y.  (2010)^14^ | 7 | 100%RIC | PR 2  SD 1  PD 4 | NA | 2004-2007 | MRD7 | NA | 4 |
| Warlick, E. D.  (2011)^15^ | NA | 100%RIC | NA | Intermediate3 advanced 7 | 2002-2008 | HLA 5/6 or 6/6 related donor match | NA | 3 |
| Jamshed,S (2011)^16^ | 6 | 100%RIC | NA | Normal 16  t(8;22)(q24;q11.2) 1 | NA | HLA-matched sibling |  | 3 |
| Einsele, H (2010)^17^ | NA | 100%MA | PR 14  SD 2  PD 2 | NA | 1995-2001 | HLA-identical  sibling17  MUD1 | NA | 5 |
| Bruno, B.  (2009)^18^ | NA | 100%NMA | CR:6  VGPR:29  PR:38 | NA | 1999-2005 | HLA–  identical sibling | NA | 4 |
| Caballero-Velazquez, T.  (2013)^19^ | 16 | 100%RIC | CR 2  PR 11  SD 1  PD 2 | High risk | 2007-2010 | RD13  UD3 | NA | 5 |

| Author(years) | N | Gender  (male/female) | Median age(range), years | Median follow-up(range),months | retrospective | conditioning regimens | strategies for GVHD prophylaxis | Source of the transplanted stem cell(peripheral blood stem cell/bone marrow) |
| --- | --- | --- | --- | --- | --- | --- | --- | --- |
| de Lavallade, H.(2008)^20^ | 19 | 15/4 | 54 (37–63) | 36 ( 21–60) | + | FLU+BU+ATG  FLU+TBI | CSA  CSA+MMF | 100%PBSC |
| Dhakal, B  (2016)^21^ | 77 | 49/28 | 53(23-69) | 50(2.3-129.3) | + | TBI  CY+FLU/TBI  FLU+MEL/TBI | MTX+ tacrolimus  MTX+CY | NA |
| Efebera, Y. A.(2010)^22^ | 51 | 27/24 | 51 (32-65) | 27(3-98) | + | FLU+MEL | Tacrolimus+MTX | PBSC41  BM10 |
| El-Cheikh, J.  (2012)^23^ | MRD23  MUD17 | MRD  13/10  MUD  10/7 | MRD  57 (40–67)  MUD  54 (39–63) | 22 (1–49) | _ | FLU+BU  FLU+TBI | CSA  CSA+MMF | PBSC37  BM3 |
| El-Cheikh, J.  (2013)^24^ | 53 | NA | 50 (28–70) | 84 (51–141) | + | FLU+BU+ATG  MEL | NA | NA |

CsA : cyclosporine-A ; MTX: methotrexate ; TT: thiotepa ;Flu: fludarabine; MEL: melphalan; Bu:Busulfan; CY:cyclophosphamide; ATG = antithymocyte globulin; GVHD = graft-versus-host disease;TBI = total-body irradiation; MTX: methotrexate;PDN: prednisone; FK506:tacrolimus; MMF: mycophenolate mofetil;

| Author(years) | Previous auto-SCT | Regimen(MA/RIC) | Status at  transplantations | Cytogenetic risk | Transplantation period | Donor type | Stage ISS | Score |
| --- | --- | --- | --- | --- | --- | --- | --- | --- |
| de Lavallade, H.(2008)^20^ | 19 | 100%RIC | PR:9  SD:9  PD:1 | NA | 2002-2004 | HLA identical sibling donor | NA | 5 |
| Dhakal, B  (2016)^21^ | 76 | 66NMA/RIC  11MA | CR 17  VGPR 6  PR 47  SD/PD 7 | high-risk27  Standard risk 50 | 2002-2013 | HLA  identical sibling 69  MUD 8 | I 27  II 21  III 28  Missing 1 | 4 |
| Efebera, Y. A.  (2010)^22^ | 36 | 100%RIC | CR 2/VGPR 3  PR 23/SD14  PD 8  Unknown/not evaluated 1 | high-risk12  Standard risk 28 | 1996-2006 | RD 40  UD 11 | NA | 5 |
| El-Cheikh, J.  (2012)^23^ | 39 | 100%RIC | MRD;  CR/VGPR 10  PR /SD 12  PD/refractory 1  MUD:  CR/VGPR 6  PR /SD 10  PD/refractory 1 | high-risk40 | 2007-2011 | MUD17  MRD23 | NA | 5 |
| El-Cheikh, J.  (2013)^24^ | 51 | 100%RIC | PR:42 | high-risk22  intermediate/low risk31 | 2000-2007 | sibling donor51 | III 46 | 3 |

| Author(years) | N | Gender  (male/female) | Median age(range),  years | Median follow-up(range),months | retrospective | conditioning regimens | strategies for GVHD prophylaxis | Source of the transplanted stem cell(peripheral blood stem cell/bone marrow) |
| --- | --- | --- | --- | --- | --- | --- | --- | --- |
| Fabre, C  （2012）^25^ | 146 | 86/60 | 51 [21-66] | 47.5(1.2-132) | + | MA:CY+TBI/BU  NMA:FLU+BU/TBI | CSA  MMF | PBSC 136  Cord blood 4  BM 6 |
| Franssen, L. E.  (2016)^26^ | First-line 58  Relapsed/  refractory  89 | First-line:37/21  Relapsed/refractory:63/26 | First-line:  53.3(35-66)  Relapsed/refractory:55.64(32-68) | 88.8 | _ | MA:  BU+FLU/CY+TBI | NA | First-line:  PBSC:56  BM1/MISSing 1  Relapsed/refractory:PBSC:56  BM7/MISSing 1: |
| Freytes, C. O.(2014)^27^ | 152 | 90/62 | 53 | 30(12–98 ) | + | MEL+TBI  FLU+BU/CY | FK506+MTX  MTX+CSA | PBSC126  BM26 |
| Gahrton, G.(2007)^28^ | 1667  PBSC1179  BM488 | PBSC:748/431  BM:288/200 | PBSC:  0-45:339  >45:840  BM:  0-45:238  >45:250 | NA | + | FLU+MEL/BU/TBI  TBI+MEL/CY  BU+CY | NA | PBSC1179  BM488 |
| Gahrton, G.(2013)^29^ | 108 | NA | 54 | 96 (47–127 ) | _ | FLU+TBI | CY+MMF | NA |
| Georges, G. E.（2007）^30^ | 24 | 19/5 | 50(29-66) | 3 years (1.2–5.5years) | _ | FLU+TBI | MMF | 100%PBSC |

| Author(years) | Previous auto-SCT | Regimen  (MA/RIC) | Status at  transplantations | Cytogenetic risk | Transplantation period | Donor type | Stage ISS | Score |
| --- | --- | --- | --- | --- | --- | --- | --- | --- |
| Fabre, C  （2012）^25^ | 146 | MA:17  NMA:129 | CR 29  VGPR 13  PR 83/MR 6  SD6/PD 7 | del(13q):40 missing:67 failures: 8 | 1998-2010 | Sibling 115  UD31 | I/II48  III27  missing71 | 5 |
| Franssen, L. E.(2016)^26^ | 147 | First-line:  MA 3/NMA 55  Semi-ablative 0 Relapsed/refractory  MA 2 /NMA 58  Semi-ablative 29 | First-line:  CR 0/ VGPR 29  PR 20  MR9 Relapsed/refractoy  CR11/VGPR32  PR 37  MR 9 | First-line: del(13q)17 Nomal25  Unknown16  Relapsed/refractoy  del(13q)18  Nomal26  Unknown45 | 2001-2014 | First-line:  Sibling55/MUD3  Relapsed/refractory:  Sibling32/MUD57 | NA | 4 |
| Freytes, C. O.  (2014)^27^ | 152 | RIC111  NMA41 | CR/PR 1  MR/SD7  PD25  Missing119 | NA | 1995-2008 | HLA-identical siblings 32  MUD 120 | NA | 5 |
| Gahrton, G.  (2007)^28^ | PBSC:486  BM:75 | RIC 648  MAC 770 | PBSC:  CR143/PR617  SD120/PD187  BM:  CR75/PR236  SD67/PD60 | NA | 1994-2003 | HLA-identical siblings | NA | 4 |
| Gahrton, G.  (2013)^29^ | 102 | 100%RIC | NA | del(13q)29 | 2001-2005 | HLA-identical sibling | NA | 3 |
| Georges, G. E.(2007)^30^ | 13 | 100%NMA | CR2  PR5  PD17 | NA | 2000-2004 | MRD23  MUD1 | NA | 5 |

| Author(years) | N | Gender  (male/female) | Median age(range), years | Median follow-up(range),months | retrospective | conditioning regimens | strategies for GVHD prophylaxis | Source of the transplanted stem cell(peripheral blood stem cell/bone marrow) |
| --- | --- | --- | --- | --- | --- | --- | --- | --- |
| Gerull, S.  (2013)^31^ | 95 | 57/38 | 47 (22–66) | 53 (7–233) | + | MEL+TBI  FLU+BU/MEL  FLU+TBI | NA | PBSC 84  BM 10  Cord blood 1 |
| Giaccone, L.  （2011）^32^ | 58 | NA | NA | 6.5years (4.2-9.4) | _ | TBI | NA | 100%PBSC |
| Karlin, L.  (2011)^33^ | 23 | 11/12 | 50 (29–59) | 27.4(1.8-61.8) | + | TBI | CSA+MMF | 100%PBSC |
| Krishnan, A  (2011)^34^ | Standard risk :189  High risk:37 | Standard risk 111/78  High risk  21/16 | Standard risk  53 (29–68)  High risk  51 (32–66) | 40(38-43) | _ | TBI | Ciclosporin+MMF | 100%PBSC |
| Kroger, N.  (2013)^35^ | 73 | 44/29 | 49 (28-64) | 72 (30-131) | _ | Mel+FLU | CSA+MMF | PBSC70  BM 3 |

| Author  (years) | Previous auto-SCT | Regimen  (MA/RIC) | Status at  transplantations | Cytogenetic risk | Transplantation period | Donor type | Stage ISS | Score |
| --- | --- | --- | --- | --- | --- | --- | --- | --- |
| Gerull, S.  (2013)^31^ | 68 | MA:32  RIC:42  NMA:21 | CR 16  VGPR 19  PR 36  SD 15  PD 9 | NA | 1988-2011 | Identical sibling 66  MUD23  MMRD 1  MMUD 4  Cord blood 1 | I:13  II:20  III:19  Unvailing:43 | 4 |
| Giaccone, L.（2011）^32^ | 58 | 100%NMA | CR:8  PR:36 | NA | 1998-2004 | HLA-identical sibling | NA | 4 |
| Karlin, L.  (2011)^33^ | 23 | 100%NMA | CR 4  VGPR 10  PR 7  Refractory2 | NA | 2002-2006 | HLA-identical siblings13  MUD10 | NA | 5 |
| Krishnan, A  (2011)^34^ | Standard risk :189  High risk:37 | 100%NMA | Standard risk  CR24/NCR22  VGPR32/PR76  MR17/SD6  Not assessable12  High risk  CR3/NCR2  VGPR7/PR14  MR3/SD4  Not assessable4 | NA | 2003-2007 | HLA-matched sibling | NA | 5 |
| Kroger, N.  (2013)^35^ | 73 | 100%RIC | CR 4/PR 57  mCR 5  SD 3  PD 4 | del13q14 :43 | 2000-2008 | HLA-identical sibling23  MUD25  MMUD25 | NA | 5 |

| Author(years) | N | Gender  (male/female) | Median age(range), years | Median follow-up(range),months | retrospective | conditioning regimens | strategies for GVHD prophylaxis | Source of the transplanted stem cell(peripheral blood stem cell/bone marrow) |
| --- | --- | --- | --- | --- | --- | --- | --- | --- |
| Kroger, N (2010)^36^ | 48 | 22/26 | 50 (34–64) | 43 | _ | MEL+FLU | ATG | PBSC44  BM4 |
| Zabelina, T. (2013)^37^ | 33 | 26/7 | 50( 36–67) | 19 (3–58) | + | BU+CY+TMI | CSA | 100%PBSC |
| Kumar, S.  (2011)^38^ | 1989-1994:343 1995-2000:376  2001-2005:488 | 1989-1994 :  194/149 1995-2000:  243/133  2001-2005:  295/193 | 1989-1994 :  44(24-59)  1995-2000:  47 (22-66)  2001-2005:  51 (24-70) | 62(3-204) | _ | MA:  CY+TBI/BU  NMA/RIC:  FLU+TBI/CY | T-cell depletion FK506+MTX/others  CsA+MTX/others | 1989-1994 :  BM 342/PBSC 1  1995-2000 :  BM196/PBSC180  2001-2005:  BM58/PBSC430 |
| Lokhorst, Hm(2012)^39^ | 122 | 71/51 | 54 (32-65) | 77(56-96) | _ | TBI | CSA+MMF | NA |
| Minnema, M.C.(2011)^40^ | 38 | 32/6 | 56 (36–68) | 2.3 years (0.2–7.2) | + | TBI+FLU+ATG | CSA+MMF | NA |

| Author  (years) | Previous auto-SCT | Regimen  (MA/RIC) | Status at  transplantations | Cytogenetic risk | Transplantation period | Donor type | Stage ISS | Score |
| --- | --- | --- | --- | --- | --- | --- | --- | --- |
| Kroger, N (2010)^36^ | 48 | 100%RIC | CR 2/PR 23  Minor remission1  SD 2/PD11  Untreated relapse7  Unknown 2 | del 13q14:17  Normal:13  Unknown:18 | 2002-2007 | MMUDdonor:17  MUD31  RD16 | NA | 5 |
| Zabelina, T. (2013)^37^ | 32 | myeloablative toxicity-reduced allograft | CR 1/PR 21  SD 3/PD 3  Untreated relapse 5 | del13q14:13  Normal:16  Unknown:4 | 2006-2010 | HLA-identical sibling6  MUD19  MMUD 8 | NA | 5 |
| Kumar, S.  (2011)^38^ | 1989-1994 :9  1995-2000 :86  2001-2005:313 | MA;  1989-1994 :324  1995-2000 :279  2001-2005:105  NMA/RIC:  1989-1994 :14  1995-2000 :86  2001-2005:344 | NA | 1989-1994  Abnormal:2 /Normal: 3  Not performed /reported :338  1995-2000  Abnormal:23/Normal:44  Not performed /reported:309  2001-2005  Abnormal33/Normal106  Not performed  /reported349 | 1989-2005 | 1989-1994 :  HLA-identical 316 Other related 11 UD 16  1995-2000 :  HLA-identical 262  Other related 19 UD 95  2001-2005:  HLA-identical322 Other related 9 UD157 | NA | 4 |
| Lokhorst, Hm(2012)^39^ | 99 | RIC:99 | CR 6  VGPR 38  PR 50  Less than PR 28 | del 13q:26  Normal:49  Unknown:47 | 2003-? | HLA-identical sibling | I:51  II 22  III 19  Unknown30 | 4 |
| Minnema, M. C.(2011)^40^ | 38 | 100%NMA | CR 3  VGPR 11  PR 23  PD 1 | NA | 2001-2008 | MUD18  HLA-identical sibling20 | NA | 4 |

| Author(years) | N | Gender  (male/female) | Median age(range), years | Median follow-up(range),months | retrospective | conditioning regimens | strategies for GVHD prophylaxis | Source of the transplanted stem cell(peripheral blood stem cell/bone marrow) |
| --- | --- | --- | --- | --- | --- | --- | --- | --- |
| Nishihori, T.  (2013)^41^ | 22 | NA | 49 (25–59) | 18 ( 2–61) | + | FLU+MEL+bortezomib | Tacrolimus+MTX  Tacrolimus+MMF | 100%PBSC |
| Nivison-Smith, I.  (2011)^42^ | 95 | 64/31 | 52 (28–63) | NA | + | FLU+MEL/TBI | CSA  CSA+MTX/MMF | PBSC89  BM6 |
| Osman, K.(2010)^43^ | 20 | 13/7 | 52.7 (37.2–68.0) | 38 | + | TBI+ATG+FLU | MMF+CSA | 100%PBSC |
| Passera, R.  (2013)^44^ | 196 | 120/76 | 51 (32-67) | 93(25 -189) | + | CY+TBI/BU  FLU+BU/TT  TBI+MEL | CSA+MTX/MMF  ATG | PBSC150  BM49 |
| Patriarca, F.  (2012)^45^ | 68 | NA | 55 (34-68) | 19(1-97) | + | FLU+CY/TBI/TT  BU+MEL | CSA+MTX  MMF  ATG | PBSC:57  BM:11 |
| Pawarode, A.  (2016)^46^ | 22 | 14/8 | 54 (45-70) | 58.7 (39.5-82) | - | Flu+BU | Tacrolimus+MTX | 100%PBSC |

| Author  (years) | Previous auto-SCT | Regimen  (MA/RIC) | Status at  transplantations | Cytogenetic risk | Transplantation period | Donor type | Stage ISS | Score |
| --- | --- | --- | --- | --- | --- | --- | --- | --- |
| Nishihori, T.  (2013)^41^ | 3 | 100%RIC | CR:10  VGPR:12 | High-risk 7 | 2007-2012 | RD9  UD 13 | NA | 5 |
| Nivison-Smith, I.  (2011)^42^ | 63 | 100%RIC | CR 7  VGPR 17  PR 33  SD 22  PD 16 | NA | 1998-2006 | HLA-identical sibling 83  UD 9 | NA | 5 |
| Osman, K.(2010)^43^ | 17 | 100%NMA | CR 9  Active 11  Refractory 6  Relapsed 3  PR 2 | del13q14:4 | 2000-2006 | MRD:14  MUD:6 | I:16  II:2  III:2 | 5 |
| Passera, R.  (2013)^44^ | 175 | MA:52  NMA:64  RIC:69 | CR:29  PR:87 | NA | 2000-2009 | UD:196 | NA | 5 |
| Patriarca, F.  (2012)^45^ | 68 | 100%NMA/RIC | NA | Standard risk 20  High risk 13 Missing 42 | 2002-2008 | HLA-matched sibling 24  MUD24  MMUD 6  Missing 14 | NA | 4 |
| Pawarode, A.  (2016)^46^ | 14 | 100%MA | NA | High risk22 | 2008-2011 | RD12  UD 10 | NA | 4 |

| Author(years) | N | Gender  (male/female) | Median age(range), years | Median follow-up(range),months | retrospective | conditioning regimens | strategies for GVHD prophylaxis | Source of the transplanted stem cell(peripheral blood stem cell/bone marrow) |
| --- | --- | --- | --- | --- | --- | --- | --- | --- |
| Ramasamy, K.(2011)^47^ | 19 | 10/9 | 53(40-62) | 24(4-105) | + | Alemtuzumab+FLU+MEL | CSA | 100%PBSC |
| Roos-Weil, D.（2011）^48^ | 143 | 85/58 | 51 (29-62) | 34(3-111) | + | NA | NA | PBSC:110  BM:29  Cord blood:4 |
| Rosinol, L.  (2015)^49^ | MA:25  RIC:33 | MAC:  19/6  RIC:  19/14 | MAC:43(23-53)  RIC:49(29-66) | MAC:  18.1 years  RIC:  5.3 years | _ | MAC:  CY+TBI/MEL/BU  BU+MEL  RIC:  FLU+TBI/MEL | MAC;  CY+MTX  T-cell depletion  CY+PDN  RIC;  CY+MTX/MMF | MAC:  BM9/PBSC:16  RIC:  PBSC:33 |
| Rosinol, L.  (2008)^50^ | 25 | NA | 52 | 5.2 years | - | FLU+MEL | CSA+MTX | NA |
| Rotta, M.  (2009)^51^ | 102 | NA | 52 (35-71) | 6.3 years | _ | TBI+FLU | CSA/FK506+MMF | 100%PBSC |

| Author  (years) | Previous auto-SCT | Regimen  (MA/RIC) | Status at  transplantations | Cytogenetic risk | Transplantation period | Donor type | Stage ISS | Score |
| --- | --- | --- | --- | --- | --- | --- | --- | --- |
| Ramasamy, K.(2011)^47^ | 15 | 100%RIC | CR:8/VGPR:4  PR:4  SD:1/PD:2 | NA | 1999-2009 | MRD:16  MUD:3 | NA | 5 |
| Roos-Weil, D.（2011）^48^ | 124 | MA:32  RIC:108 | CR+VGPR:27  PR:92  SD+PD:23 |  | 1999-2008 | Identical sibling 97  MUD:34  MMUD:12 | NA | 3 |
| Rosinol, L.  (2015)^49^ | NA | MA:25  RIC:33 | NA | NA | 1986-2012 | MAC:  identical sibling25  RIC :  identical sibling24  MUD5/MMUD4 | MAC:  I 5  II 4  III 4  Unknown12  RIC:  I 9  II 5  III 4  Unknown15 | 4 |
| Rosinol, L.  (2008)^50^ | 25 | 100%RIC | NA | NA | 1999-2004 | HLA-identical sibling | I :11  II :9  III :1 | 3 |
| Rotta, M.  (2009)^51^ | 102 | 100%NMA | CR:19/VGPR:24  PR:41  refractory /SD:16  PD:2 | NA | 1998-2005 | HLA-identical sibling | NA | 5 |

| Author(years) | N | Gender  (male/female) | Median age(range), years | Median follow-up(range),months | retrospective | conditioning regimens | strategies for GVHD prophylaxis | Source of the transplanted stem cell(peripheral blood stem cell/bone marrow) |
| --- | --- | --- | --- | --- | --- | --- | --- | --- |
| Sahebi, F.  (2015)^52^ | Auto–allo:517  Early RIC:173 | Auto–allo:  313/204  Early RIC:  89/84  备注：The early RIC allograft group received RIC allograft as first transplant | Auto–allo:53 (21–72)  Early RIC:51 (31–77) | 93 | + | TBI  FLU+MEL | NA | Auto–allo:  PBSC:441  BM27  Early RIC:  PBSC149  BM23 |
| Schilling, G.  （2008）^53^ | 101 | 59/42 | 52 ( 28–68) | 33 (3–73) | _ | MEL+FLU+ATG | NA | PBSC97  BM4 |
| Shimoni, A.  (2010)^54^ | 50 | 29/21 | 53(32-64) | 6.4years (5-7.9 ) | + | FLU+MEL | CSA+MTX | 100%PBSC |
| van Dorp, S.  (2007)^55^ | 59  frst-line treatment：36  salvage therapy：23 | 42/17 | 55 (35 - 67) | 25.2(6.8-54.6) | + | TBI+FLU | CSA+MMF | NA |
| Smith, E.  (2016)^56^ | 44 | 31/13 | 55.5 (32 - 68 ) | 24.8  (11.2 - 81.2). | + | BU+MEL+FLU+ATG | NA | 100%PBSC |
| Costa, L. J.  (2009)^57^ | 33 | 19/14 | 48 (33–61) | 25.6 | + | MA:CY+BU/TBI  MEL+TBI  RIC:FLU+MEL/TBI/CY | MTX+CSA/FK506 | PBSC:21  BM:12 |
| Mir, M. A.  (2015)^58^ | 66 | 42/24 | 48 (28-61) | NA | + | FLU+CY/TBI/MEL  CY+BU/TBI | CSA  FK506 | NA |
| Nair, A. P.  (2017)^59^ | Upfront:29  Deferred:30 | Upfront tandem ASCT/NMA HSCT:  13/16  Deferred tandem ASCT/NMA HSCT:  23/7 | Upfront:  52 (22–66)  Deferred:  56.5 (43–67) | 48,.3 | + | FLU+TBI | CSA+MMF | 100%PBSC |
| Kawamura, K.(2016)^60^ | 89 | 52/37 | 49 (19–66) | 1429 days  (8–5594 days) | - | NA | NA | PBSC:43  BM:31  cord blood:15 |
| Kikuchi, T.  (2015)^61^ | 23 | 10/13 | 46 (33–54) | 73.2(46–158.9) | + | FLU+MEL | CSA/FK506+MTX | PBSC:1  BM:22 |

| Author  (years) | Previous auto-SCT | Regimen  (MA/RIC) | Status at  transplantations | Cytogenetic risk | Transplantation period | Donor type | Stage ISS | Score |
| --- | --- | --- | --- | --- | --- | --- | --- | --- |
| Sahebi, F.  (2015)^52^ | Auto-allo:517 | Auto-allo:  MA38  RIC:434  Early RIC:RIC173 | Auto–allo:CR 45  PR 349 /Other 109  Early RIC:CR 26  PR 106 /Other 33 | NA | 1996-2013 | Auto–allo:MRD:410  MUD30/Others32  Early RIC:MRD:144  MUD9/Others20 | NA | 4 |
| Schilling, G.  （2008）^53^ | 97 | 100%RIC | CR 3/PR 69  MR 1/SD2  PD26 | NA | NA | MRD93  MMUD8 | NA | 4 |
| Shimoni, A.  (2010)^54^ | 47 | 100%RIC | PR/CR 30  SD/PD 20 | NA | 2001-2004 | RD:27  UD:23 | NA | 5 |
| van Dorp, S.(2007)^55^ | NA | 100%NMA | CR:9  No CR:50 | del13q 29 | 2001-2005 | MRD:48  MUD:11 | NA | 4 |
| Smith, E.  (2016)^56^ | 44 | NA | VGPR/CR:23  PR:21 | high-risk29 | 2007-2013 | Identical sibling:14  MUD:18  MMUD12 | NA | 4 |
| Costa, L. J.  (2009)^57^ | 19 | MA:17  RIC:16 | CR6/PR13  PD12/ Unknown2 | NA | 1991-2006 | MRD25  MMRD2  MUD6 | NA | 5 |
| Mir, M. A.  (2015)^58^ | 50 | MA:47  RIC:19 | Relapsed/refractory/  Progression 29  PR/CR 37 | NA | 1993-2013 | matched sibling donors46  UD20 | NA | 4 |
| Nair, A. P.  (2017)^59^ | 59 | 100%NMA | NA | NA | 2008-2015 | Upfront:  UD18/RD11  Deferred:  UD18/RD12 | NA | 4 |
| Kawamura, K.(2016)^60^ | 89 | MA:11  RIC:77  Unclassifiable:1 | CR12/VGPR24  PR43/MR3  SD4/PD3 | NA | 1998-2012 | UD:36  RD:53 | I:28  II:22  III:27  Data missing12 | 3 |
| Kikuchi, T.  (2015)^61^ | 21 | 100%RIC | CR4/VGPR3  PR10  SD1/PD5 | NA | 2001-2011 | Identical sibling:4  HLA-mismatched sibling:1  MUD:14/MMUD:4 | I:7  II:6  III:5  Data missing5 | 5 |

CsA : cyclosporine-A ; MTX: methotrexate ; TT: thiotepa ;Flu: fludarabine; MEL: melphalan; Bu: Busulfan; CY: cyclophosphamide; ATG: antithymocyte globulin; ISS: International Staging System ;TBI : total-body irradiation; TMI: total marrow irradiation; MTX: methotrexate; PDN: prednisone; FK506:tacrolimus; MMF: mycophenolate mofetil; Bz: Bortezomib; MRD: matched related donor; MUD :matched unrelated donor; MMUD: mismatched unrelated donor; MMRD: mismatched related donor ; RD: related donor; UD: unrelated donor ; MR: Minimal response; VGPR :very good partial response; NCR: Near complete response; mCR: molecular remission ; CR: complete response;

PR: partial response PD: progressive disease; SD: stable disease; del(13q): Chromosome 13 deletion; NA :not available; RIC = reduced intensity conditioning; NMA: nonmyeloablative allogeneic stem cell;

1 Majolino, I. *et al.* Reduced intensity conditioning with thiotepa, fludarabine, and melphalan is effective in advanced multiple myeloma. *Leukemia and Lymphoma* **48**, 759-766 (2007).

2 Novitzky, N., Thomas, V. & du Toit, C. Prevention of graft vs. host disease with alemtuzumab 'in the bag' decreases early toxicity of stem cell transplantation and in multiple myeloma is associated with improved long-term outcome. *Cytotherapy* **10**, 45-53, doi:10.1080/14653240701732771 (2008).

3 Ringden, O. *et al.* Effect of acute and chronic GVHD on relapse and survival after reduced-intensity conditioning allogeneic transplantation for myeloma. *Bone marrow transplantation* **47**, 831-837, doi:10.1038/bmt.2011.192 (2012).

4 Sahebi, F. *et al.* Late relapses following reduced intensity allogeneic transplantation in patients with multiple myeloma: a long-term follow-up study. *British journal of haematology* **160**, 199-206, doi:10.1111/bjh.12123 (2013).

5 Schmidt-Hieber, M. *et al.* Reduced-toxicity conditioning with fludarabine and treosulfan prior to allogeneic stem cell transplantation in multiple myeloma. *Bone marrow transplantation* **39**, 389-396, doi:10.1038/sj.bmt.1705605 (2007).

6 Ahmad, I. *et al.* Favorable long-term outcome of patients with multiple myeloma using a frontline tandem approach with autologous and non-myeloablative allogeneic transplantation. *Bone marrow transplantation* **51**, 529-535, doi:10.1038/bmt.2015.319 (2016).

7 Auner, H. W. *et al.* Reduced intensity-conditioned allogeneic stem cell transplantation for multiple myeloma relapsing or progressing after autologous transplantation: A study by the European group for blood and marrow transplantation. *Bone Marrow Transplantation* **48**, 1395-1400 (2013).

8 Bashir, Q. *et al.* Predictors of prolonged survival after allogeneic hematopoietic stem cell transplantation for multiple myeloma. *American journal of hematology* **87**, 272-276, doi:10.1002/ajh.22273 (2012).

9 Beaussant, Y. *et al.* Hematopoietic Stem Cell Transplantation in Multiple Myeloma: A Retrospective Study of the Societe Francaise de Greffe de Moelle et de Therapie Cellulaire (SFGM-TC). *Biology of Blood and Marrow Transplantation* **21**, 1452-1459 (2015).

10 Bjorkstrand, B. *et al.* Tandem autologous/reduced-intensity conditioning allogeneic stem-cell transplantation versus autologous transplantation in myeloma: long-term follow-up. *Journal of clinical oncology : official journal of the American Society of Clinical Oncology* **29**, 3016-3022, doi:10.1200/jco.2010.32.7312 (2011).

11 Bruno, B. *et al.* A comparison of allografting with autografting for newly diagnosed myeloma. *The New England journal of medicine* **356**, 1110-1120, doi:10.1056/NEJMoa065464 (2007).

12 Bruno, B. *et al.* Unrelated donor haematopoietic cell transplantation after non-myeloablative conditioning for patients with high-risk multiple myeloma. *European journal of haematology* **78**, 330-337, doi:10.1111/j.1600-0609.2007.00816.x (2007).

13 Crawley, C. *et al.* Reduced-intensity conditioning for myeloma: lower nonrelapse mortality but higher relapse rates compared with myeloablative conditioning. *Blood* **109**, 3588-3594, doi:10.1182/blood-2006-07-036848 (2007).

14 Hong, J. Y. *et al.* Feasibility of second hematopoietic stem cell transplantation using reduced-intensity conditioning with fludarabine and melphalan after a failed autologous hematopoietic stem cell transplantation. *Transplantation Proceedings* **42**, 3723-3728 (2010).

15 Warlick, E. D. *et al.* Reduced-intensity conditioning followed by related allografts in hematologic malignancies: long-term outcomes most successful in indolent and aggressive Non-Hodgkin lymphomas. *Biology of Blood and Marrow Transplantation* **17**, 1025-1032 (2011).

16 Jamshed, S. *et al.* EPOCH-F: A novel salvage regimen for multiple myeloma before reduced-intensity allogeneic hematopoietic SCT. *Bone marrow transplantation* **46**, 676-681 (2011). <<http://onlinelibrary.wiley.com/o/cochrane/clcentral/articles/298/CN-00891298/frame.html>

<http://www.nature.com/bmt/journal/v46/n5/pdf/bmt2010173a.pdf>>.

17 Kroger, N. *et al.* Long-term follow-up of an intensified myeloablative conditioning regimen with in vivo T cell depletion followed by allografting in patients with advanced multiple myeloma. *Biology of blood and marrow transplantation : journal of the American Society for Blood and Marrow Transplantation* **16**, 861-864, doi:10.1016/j.bbmt.2010.01.018 (2010).

18 Bruno, B. *et al.* Nonmyeloablative allografting for newly diagnosed multiple myeloma: the experience of the Gruppo Italiano Trapianti di Midollo. *Blood* **113**, 3375-3382, doi:10.1182/blood-2008-07-167379 (2009).

19 Caballero-Velazquez, T. *et al.* Phase II clinical trial for the evaluation of bortezomib within the reduced intensity conditioning regimen (RIC) and post-allogeneic transplantation for high-risk myeloma patients. *British journal of haematology* **162**, 474-482, doi:10.1111/bjh.12410 (2013).

20 de Lavallade, H. *et al.* Reduced-intensity conditioning allogeneic SCT as salvage treatment for relapsed multiple myeloma. *Bone marrow transplantation* **41**, 953-960, doi:10.1038/bmt.2008.22 (2008).

21 Dhakal, B. *et al.* Allogeneic Hematopoietic Cell Transplantation in Multiple Myeloma: Impact of Disease Risk and Post Allograft Minimal Residual Disease on Survival. *Clinical lymphoma, myeloma & leukemia* **16**, 379-386 (2016). <<http://onlinelibrary.wiley.com/o/cochrane/clcentral/articles/587/CN-01165587/frame.html>

<http://ac.els-cdn.com/S2152265016300131/1-s2.0-S2152265016300131-main.pdf?_tid=df5e57e2-37a3-11e7-b820-00000aab0f6b&acdnat=1494656442_713d55cb5b643a3fc30bb7fec09435f7>>.

22 Efebera, Y. A. *et al.* Reduced-Intensity Allogeneic Hematopoietic Stem Cell Transplantation for Relapsed Multiple Myeloma. *Biology of Blood and Marrow Transplantation* **16**, 1122-1129 (2010).

23 El-Cheikh, J. *et al.* Comparable outcomes between unrelated and related donors after reduced-intensity conditioning allogeneic hematopoietic stem cell transplantation in patients with high-risk multiple myeloma. *European Journal of Haematology* **88**, 497-503 (2012).

24 El-Cheikh, J. *et al.* Long-term outcome after allogeneic stem-cell transplantation with reduced-intensity conditioning in patients with multiple myeloma. *American Journal of Hematology* **88**, 370-374 (2013).

25 Fabre, C. *et al.* Younger donor's age and upfront tandem are two independent prognostic factors for survival in multiple myeloma patients treated by tandem autologous-allogeneic stem cell transplantation: a retrospective study from the Societe Francaise de Greffe de Moelle et de Therapie Cellulaire (SFGM-TC). *Haematologica* **97**, 482-490, doi:10.3324/haematol.2011.049742 (2012).

26 Franssen, L. E. *et al.* Outcome of allogeneic transplantation in newly diagnosed and relapsed/refractory multiple myeloma: long-term follow-up in a single institution. *European journal of haematology* **97**, 479-488, doi:10.1111/ejh.12758 (2016).

27 Freytes, C. O. *et al.* Second transplants for multiple myeloma relapsing after a previous autotransplant-reduced-intensity allogeneic vs autologous transplantation. *Bone marrow transplantation* **49**, 416-421, doi:10.1038/bmt.2013.187 (2014).

28 Gahrton, G. *et al.* Peripheral blood or bone marrow cells in reduced-intensity or myeloablative conditioning allogeneic HLA identical sibling donor transplantation for multiple myeloma. *Haematologica* **92**, 1513-1518, doi:10.3324/haematol.11353 (2007).

29 Gahrton, G. *et al.* Autologous/reduced-intensity allogeneic stem cell transplantation vs autologous transplantation in multiple myeloma: long-term results of the EBMT-NMAM2000 study. *Blood* **121**, 5055-5063, doi:10.1182/blood-2012-11-469452 (2013).

30 Georges, G. E. *et al.* Nonmyeloablative unrelated donor hematopoietic cell transplantation to treat patients with poor-risk, relapsed, or refractory multiple myeloma. *Biology of blood and marrow transplantation : journal of the American Society for Blood and Marrow Transplantation* **13**, 423-432, doi:10.1016/j.bbmt.2006.11.011 (2007).

31 Gerull, S. *et al.* Allo-SCT for multiple myeloma in the era of novel agents: a retrospective study on behalf of Swiss Blood SCT. *Bone marrow transplantation* **48**, 408-413, doi:10.1038/bmt.2012.167 (2013).

32 Giaccone, L. *et al.* Long-term follow-up of a comparison of nonmyeloablative allografting with autografting for newly diagnosed myeloma. *Blood* **117**, 6721-6727, doi:10.1182/blood-2011-03-339945 (2011).

33 Karlin, L. *et al.* Tandem autologous non-myeloablative allogeneic transplantation in patients with multiple myeloma relapsing after a first high dose therapy. *Bone marrow transplantation* **46**, 250-256, doi:10.1038/bmt.2010.90 (2011).

34 Krishnan, A. *et al.* Autologous haemopoietic stem-cell transplantation followed by allogeneic or autologous haemopoietic stem-cell transplantation in patients with multiple myeloma (BMT CTN 0102): a phase 3 biological assignment trial. *The Lancet. Oncology* **12**, 1195-1203 (2011). <<http://onlinelibrary.wiley.com/o/cochrane/clcentral/articles/504/CN-00805504/frame.html>

<http://ac.els-cdn.com/S1470204511702431/1-s2.0-S1470204511702431-main.pdf?_tid=190c0afc-37a4-11e7-bb52-00000aacb35e&acdnat=1494656539_84eaeddfc36ab4ba83517a7e12017653>>.

35 Kroger, N. *et al.* Impact of high-risk cytogenetics and achievement of molecular remission on long-term freedom from disease after autologous-allogeneic tandem transplantation in patients with multiple myeloma. *Biology of blood and marrow transplantation : journal of the American Society for Blood and Marrow Transplantation* **19**, 398-404, doi:10.1016/j.bbmt.2012.10.008 (2013).

36 Kroger, N. *et al.* Unrelated stem cell transplantation after reduced intensity conditioning for patients with multiple myeloma relapsing after autologous transplantation. *British journal of haematology* **148**, 323-331, doi:10.1111/j.1365-2141.2009.07984.x (2010).

37 Zabelina, T. *et al.* Toxicity-reduced, myeloablative allograft followed by lenalidomide maintenance as salvage therapy for refractory/relapsed myeloma patients. *Bone marrow transplantation* **48**, 403-407, doi:10.1038/bmt.2012.142 (2013).

38 Kumar, S. *et al.* Trends in allogeneic stem cell transplantation for multiple myeloma: a CIBMTR analysis. *Blood* **118**, 1979-1988, doi:10.1182/blood-2011-02-337329 (2011).

39 Lokhorst, H. *et al.* Donor versus no-donor comparison of newly diagnosed myeloma patients included in the HOVON-50 multiple myeloma study. *Blood* **119**, 6219-6225; quiz 6399 (2012). <<http://onlinelibrary.wiley.com/o/cochrane/clcentral/articles/088/CN-00970088/frame.html>

<http://www.bloodjournal.org/content/bloodjournal/119/26/6219.full.pdf>>.

40 Minnema, M. C. *et al.* Prognostic factors and outcome in relapsed multiple myeloma after nonmyeloablative allo-SCT: a single center experience. *Bone marrow transplantation* **46**, 244-249, doi:10.1038/bmt.2010.101 (2011).

41 Nishihori, T. *et al.* Allogeneic hematopoietic cell transplantation for consolidation of VGPR or CR for newly diagnosed multiple myeloma. *Bone marrow transplantation* **48**, 1179-1184, doi:10.1038/bmt.2013.37 (2013).

42 Nivison-Smith, I. *et al.* Allogeneic hematopoietic cell transplant for multiple myeloma using reduced intensity conditioning therapy, 1998-2006: factors associated with improved survival outcome. *Leukemia & lymphoma* **52**, 1727-1735, doi:10.3109/10428194.2011.582201 (2011).

43 Osman, K. *et al.* Non-myeloablative conditioning and allogeneic transplantation for multiple myeloma. *American journal of hematology* **85**, 249-254, doi:10.1002/ajh.21633 (2010).

44 Passera, R. *et al.* Allogeneic hematopoietic cell transplantation from unrelated donors in multiple myeloma: study from the Italian Bone Marrow Donor Registry. *Biology of blood and marrow transplantation : journal of the American Society for Blood and Marrow Transplantation* **19**, 940-948, doi:10.1016/j.bbmt.2013.03.012 (2013).

45 Patriarca, F. *et al.* Allogeneic stem cell transplantation in multiple myeloma relapsed after autograft: A multicenter retrospective study based on donor availability. *Biology of Blood and Marrow Transplantation* **18**, 617-626 (2012).

46 Pawarode, A. *et al.* Reducing Treatment-Related Mortality Did Not Improve Outcomes of Allogeneic Myeloablative Hematopoietic Cell Transplantation for High-Risk Multiple Myeloma: A University of Michigan Prospective Series. *Biology of Blood and Marrow Transplantation* **22**, 54-60 (2016).

47 Ramasamy, K. *et al.* Alemtuzumab-based reduced-intensity conditioning allogeneic transplantation for myeloma and plasma cell leukemia - a single-institution experience. *Clinical lymphoma, myeloma & leukemia* **11**, 242-245, doi:10.1016/j.clml.2011.03.004 (2011).

48 Roos-Weil, D. *et al.* Impact of genetic abnormalities after allogeneic stem cell transplantation in multiple myeloma: a report of the Societe Francaise de Greffe de Moelle et de Therapie Cellulaire. *Haematologica* **96**, 1504-1511, doi:10.3324/haematol.2011.042713 (2011).

49 Rosinol, L. *et al.* Allogeneic hematopoietic SCT in multiple myeloma: Long-term results from a single institution. *Bone Marrow Transplantation* **50**, 658-662 (2015).

50 Rosinol, L. *et al.* A prospective PETHEMA study of tandem autologous transplantation versus autograft followed by reduced-intensity conditioning allogeneic transplantation in newly diagnosed multiple myeloma. *Blood* **112**, 3591-3593, doi:10.1182/blood-2008-02-141598 (2008).

51 Rotta, M. *et al.* Long-term outcome of patients with multiple myeloma after autologous hematopoietic cell transplantation and nonmyeloablative allografting. *Blood* **113**, 3383-3391, doi:10.1182/blood-2008-07-170746 (2009).

52 Sahebi, F. *et al.* Comparison of upfront tandem autologous-allogeneic transplantation versus reduced intensity allogeneic transplantation for multiple myeloma. *Bone marrow transplantation* **50**, 802-807, doi:10.1038/bmt.2015.45 (2015).

53 Schilling, G. *et al.* Impact of genetic abnormalities on survival after allogeneic hematopoietic stem cell transplantation in multiple myeloma. *Leukemia* **22**, 1250-1255, doi:10.1038/leu.2008.88 (2008).

54 Shimoni, A. *et al.* Allogenic hematopoietic stem-cell transplantation with reduced-intensity conditioning in patients with refractory and recurrent multiple myeloma: long-term follow-up. *Cancer* **116**, 3621-3630, doi:10.1002/cncr.25228 (2010).

55 van Dorp, S. *et al.* Single-centre experience with nonmyeloablative allogeneic stem cell transplantation in patients with multiple myeloma: Prolonged remissions induced. *Netherlands Journal of Medicine* **65**, 178-184 (2007).

56 Smith, E. *et al.* CD34-Selected Allogeneic Hematopoietic Stem Cell Transplantation for Patients with Relapsed, High-Risk Multiple Myeloma. *Biology of blood and marrow transplantation : journal of the American Society for Blood and Marrow Transplantation* **22**, 258-267, doi:10.1016/j.bbmt.2015.08.025 (2016).

57 Costa, L. J. *et al.* Factors associated with favorable outcome after allogeneic hematopoietic stem cell transplantation for multiple myeloma. *Leukemia & lymphoma* **50**, 781-787, doi:10.1080/10428190902803644 (2009).

58 Mir, M. A. *et al.* Trends and outcomes in allogeneic hematopoietic stem cell transplant for multiple myeloma at Mayo Clinic. *Clinical lymphoma, myeloma & leukemia* **15**, 349-357.e342, doi:10.1016/j.clml.2015.03.016 (2015).

59 Nair, A. P. *et al.* Adverse impact of high donor CD3+ cell dose on outcome following tandem auto-NMA allogeneic transplantation for high-risk myeloma. *Bone Marrow Transplantation.* **20** (2017).

60 Kawamura, K. *et al.* Tandem autologous versus autologous/allogeneic transplantation for multiple myeloma: propensity score analysis. *Leukemia and Lymphoma* **57**, 2077-2083 (2016).

61 Kikuchi, T. *et al.* Outcome of reduced-intensity allogeneic hematopoietic stem cell transplantation for multiple myeloma. *International Journal of Hematology* **102**, 670-677 (2015).
