# Supplementary material for: Allogeneic stem-cell transplantation for multiple myeloma: a systematic review and meta-analysis from 2007 to 2017
Source: Cancer Cell Int. 2018 Apr 23;18:62. doi: 10.1186/s12935-018-0553-8 (PMC5913895; doi:10.1186/s12935-018-0553-8)
Supplement: Supplementary file 2 — Additional file 2: Table S2. Patients’ transplant outcomes of individual clinical trials. [file 12935_2018_553_MOESM2_ESM.docx]

| Author  (years) | N | OS | DFS/EFS/PFS | TRM/NRM | GVHD | RR | Death and Death cause |
| --- | --- | --- | --- | --- | --- | --- | --- |
| Majolino, I.(2007)^1^ | 53 | 24(3years) | 20(3years) | 7 | aGVHD(1)6  aGVHD(2)21  aGVHD(3)2  aGVHD(4)1  Total(aGVHD)53  ext. cGVHD:14  lim. cGVHD:15  Total(cGVHD)45 | 17 | 21  In 8 the death was due to causes unrelated to the disease itself. |
| Novitzky, N.(2008)^2^ | 22 | 12(3years) | 11(3years) | 2(100days)  4(1years) | aGVHD:2  lim. cGVHD:2 | 10 | 8  Relapse3,TRM4,unkown1 |
| Ringden, O.(2012)^3^ | 177 | 133(1years)  67(5years) | 113(1years)  39(5years) | 27(1years)  44(5years) | aGVHD(1-4)74  aGVHD(2-4)53  cGVHD(1years)80  cGVHD(5years)104  ext. cGVHD:104 | 39(1years)  92(5years) | 14(100days)  The most common cause of death was relapsed or progressive MM in 33% patients, followed by infections and organ failure. |
| Sahebi, F.(2013)^4^ | 60 | 37(5years)  36(7years) | 22(5years)  19(7years) | 5(1years)  7(5years)  7(7years) | aGVHD(2-4)22  aGVHD(3-4)5  ext. cGVHD43  lim. cGVHD9  Total(cGVHD)58 | 38  32(5years)  35(7years) | 30  23 of disease progression or relapse, and seven from transplant-related complications |
| Schmidt-Hieber, M.(2007)^5^ | 34 | 24(1years)  20(2years) | 13(1years)  5(2years) | 6 (1years)  Total(1years)24  3 (100days)  Total(100days)31 | aGVHD(1)11  aGVHD(2)1  aGVHD(3)7  aGVHD(4)3  Total(aGVHD):33  ext. cGVHD:4  lim. cGVHD:7  Total(cGVHD)28 | 20 | 24  Relapse2，TRM6，unkown16 |

| Author  (years) | N | OS | DFS/EFS/PFS | TRM/NRM | GVHD | RR | Death and Death cause |
| --- | --- | --- | --- | --- | --- | --- | --- |
| Ahmad, I.  （2016）^6^ | 92 | 57(10years) | 38(10years) | 9(10years) | aGVHD(2-4):8  aGVHD(3-4):3  ext. cGVHD:73 | 45 | 19  TRM10, refractory MM9 |
| Auner, H. W.(2013)^7^ | 401 | 24.7 months (95% confidence interval, 18.7–30.7) | 9.6months (95% confidence interval, 7.3–11.8) | 42(3months)  86(1years)  114(3years) | aGVHD(1-4)188  aGVHD(2-4)125  Total(aGVHD)379  ext. cGVHD:92  lim. cGVHD:75  Total(cGVHD)345 | NA | NA |
| Bashir, Q.(2012)^8^ | 149 | 31(5years) | 22 (5years) | 25(100days)  70(5years) | aGVHD(1-4)52  aGVHD(2-4)25  ext. cGVHD:69  lim. cGVHD:69 | NA | 101  The most common causes of death were recurrent disease52(35%), aGVHD15(10%), cGVHD15 (10%), infection10(7%), and multiorgan failure 9(6%). |
| Beaussant, Y.(2015)^9^ | RIC:397  MAC:49 | RIC:  236(2years)  150(5years)  MAC:  33(2years)  26(5years) | RIC:  141(2years)  77(5years)  MAC:  25(2years)  13(5years) | RIC:  98(2years)  132(5years)  MAC:  11(2years)  22(5years) | aGVHD(1)63  aGVHD(2-4)162  cGVHD:221  ext. cGVHD:107  lim. cGVHD:103 | NA | RIC：261  Relapse or progression of original disease 145，TRM100, Secondary malignancy 7, Other 6  MAC：27  Relapse or progression of original disease 15，TRM9, Other 1， |
| Bjorkstrand, B.(2011)^10^ | 108 | 70(5years) | 38(5years) | 13(2years)  17(5years) | aGVHD(1)10  aGVHD(2)8  aGVHD(3)8  aGVHD(4)2  Total(aGVHD):91  Total(cGVHD)91  ext. cGVHD:21  lim. cGVHD:28 | 53(5years) | NA |
| Bruno, B(2007)^11^ | 58 | NA | NA | NA | aGVHD(2-4)25  aGVHD(4)2  ext. cGVHD:21 | NA | 12  4 from disease progression, 6 from transplant-related causes, and 2 from lung cancer. |

| Author  (years) | N | OS | DFS/EFS/PFS | TRM/NRM | GVHD | RR | Death and Death cause |
| --- | --- | --- | --- | --- | --- | --- | --- |
| Sorasio, R.(2007)^12^ | 22 | 13(2years) | 14(2years) | 5(1years)  6(2years) | aGVHD(2-3)：9  aGVHD(4):1  ext. cGVHD:11  lim. cGVHD:4  Total(cGVHD):18 | NA | 9  Six patients died of TRM and three of disease progression |
| Crawley, C. (2007)^13^ | RIC320  MAC196 | RIC:122(3years)  MAC:100(3years) | RIC:60(3years)  MAC:68(3years) | RIC:77(2years)  MAC:73(2years) | RIC：aGVHD(2-4)114  MAC:aGVHD(2-4)90  Total(cGVHD)329  ext. cGVHD:74  lim. cGVHD:89 | RIC:173  MAC:53 | NA |
| Hong, J. Y.(2010)^14^ | 7 | 2 | NA | NA | aGVHD(2)1  aGVHD(4)1  ext. cGVHD:1 | NA | 5 |
| Warlick, E. D.(2011)^15^ | 10 | 4(1years)  1(4years) | 2(1years)  1(4years) | 3(1years) | NA | 4(1years)  4(4years) | NA |
| Jamshed,S(2011)^16^ | 22 | 46.1 months  Total 20 | NA | 1(100days)  6(5years)  Total 20 | aGVHD(2-4)10  aGVHD(4)1  Total(aGVHD)19  Total(cGVHD)18  cGVHD:9 | NA | 10 Four patients died from progressive disease, whereas four died from infectious complications, one from acute GVHD and one from gastrointestinal hemorrhage related to transplant |
| Einsele, H. (2010)^17^ | 18 | 9(12years) | 6(5years) | 3 | aGVHD(1)4  aGVHD(2)6  aGVHD(4)1  Total(aGVHD)16  ext. cGVHD:3  lim. cGVHD:4  Total(cGVHD)15 | 4 | 8  Progressed/ Relapsed7 |
| Bruno, B.(2009)^18^ | 96 | NA | EFS was 2.9 years (range, 2.4-4.3 | NA | aGVHD(2-4)36  aGVHD(4)3  Total(cGVHD)94  ext. cGVHD:47 | NA | NA |
| Caballero-Velazquez, T.(2013)^19^ | 16 | 7(3years) | 7(258 days) | 4(3years) | aGVHD(1)3  aGVHD(2)3  aGVHD(3)4  aGVHD(4)0 | 10 | 10  six due to relapse and 4 due to NRM |

| Author(years) | N | OS | DFS/EFS/PFS | TRM/NRM | GVHD | RR | Death and Death cause |
| --- | --- | --- | --- | --- | --- | --- | --- |
| de Lavallade, H.(2008)^20^ | 19 | 18(6months)  10(3years) | 9(3years) | 6  Total 18 | aGVHD(2-4)10  aGVHD(3-4)6  Total(aGVHD)18  Total(cGVHD)14  ext. cGVHD:8 | NA | 6  Total 18  Three patients died from aGVHD, one from cGVHD, one from infection and one from pulmonary embolism. |
| Dhakal, B(2016)^21^ | 77 | 64(1years)  49(3years) | 56(1years)  36(3years) | 10(1years) | aGVHD(2)14  aGVHD(3)5  aGVHD(4)3  cGVHD:29 | 2(100days)  11(1years) | NA |
| Efebera, Y. A.(2010)^22^ | 51 | 16(2years) | 10(2years) | 6(100days)  13(1years) | aGVHD(2-4)14  aGVHD(2)8  aGVHD(3-4)6  cGVHD:24  lim. cGVHD:12 | 25(2years) | 39  Disease recurrence 22  Acute or chronic GVHD 10  Infection 3  Other 4 |
| El-Cheikh, J.(2012)^23^ | MRD23  MUD17 | MRD:  15(2years)  MUD:  10(2years) | MRD:  10(2years)  MUD:  7(2years) | MRD:  5(2years)  MUD:  2(2years) | MRD:  aGVHD(2-4)4  aGVHD(2-3)3  aGVHD(4)1  cGVHD:9  ext. cGVHD:4  lim. cGVHD:5  MUD: aGVHD(2-4)8  aGVHD(2-3)8  aGVHD(4)0  cGVHD:4  ext. cGVHD:3  lim. cGVHD:1 | MRD:9  MUD:8 | MRD:9  TRM 5 ,Disease 4  MUD:8  TRM 2 ,Disease 6 |
| El-Cheikh, J.(2013)^24^ | 53 | 28(5years)  17(10years) | 14(5years)  13(10years) | 9(1years)  10(2years) | aGVHD(2-4)20  cGVHD:31 | NA | NA |

| Author(years) | N | OS | DFS/EFS/PFS | TRM/NRM | GVHD | RR | Death and Death cause |
| --- | --- | --- | --- | --- | --- | --- | --- |
| Fabre, C(2012)^25^ | 146 | 70(4years) | 39(4years) | 9(100days)  22(1years) | aGVHD(1)14  aGVHD(2)23  aGVHD(3)12  aGVHD(4)6  Total(cGVHD)141  ext. cGVHD:26  lim. cGVHD:17 | 66 | 69  Infection 21 aGVHD6 , cGVHD1, Other 4  Veno-occlusive disease1  Relapse/progression 31  Other cause (second cancer) 5 |
| Franssen, L. E.(2016)^26^ | First-line 58  Relapsed/  refractory  89 | First-line:  Relapsed/  Refractory:  28.7 months (95% CI: 16.4–41.0) | First-line: 30.2 months (95% CI: 21.4–39.0)  Relapsed/  Refractory:  8.0 months (95% CI: 6.4–9.7) | First-line :  9(10years)  Relapsed/refractory  17(10years) | First-line:  aGVHD(2-4)29  ext. cGVHD:23  lim. cGVHD:6  Relapsed/Refractory:  aGVHD(2-4)27  ext. cGVHD:21  lim. cGVHD:12 | First-line :31  Relapsed/  Refractory98 | NA |
| Freytes, C. O.  (2014)^27^ | 152 | 30(3years) | 9(3years) | 20(1years)  21(3years) | NA | NA | NA |
| Gahrton, G.(2007)^28^ | 1667  PBSC1179  BM488 | PBSC: 389(5years)  354(7years)  BM  185(5years)  176(7years) | NA | PBSC+RIC:130(100days)  PBSC+MA200(100days)  BM+RIC29(100days)  BM+MA88(100days) | PBSC:  aGVHD(2-4)409  cGVHD:637  ext. cGVHD:295  BM:  aGVHD(2-4)193  cGVHD:202  ext. cGVHD:113 | NA | NA |
| Gahrton, G.(2013)^29^ | 108 | 69(5years)  53(8years) | 36(5years)  24(8years) | 14(3years)  19(8years) | aGVHD(1)10  aGVHD(2)8  aGVHD(3)8  aGVHD(4)2  Total(aGVHD)92  Total(cGVHD)92  ext. cGVHD:21  lim. cGVHD:29 | 56(5years)  64(8years) | NA |
| Georges,G.E.(2007)^30^ | 24 | 15(3years) | 8(3years) | 1(100days)  5(3years) | aGVHD(1)0  aGVHD(2)13  aGVHD(3)3  aGVHD(4)0  cGVHD:18 | NA | NA |

| Author(years) | N | OS | DFS/EFS/PFS | TRM/NRM | GVHD | RR | Death and Death cause |
| --- | --- | --- | --- | --- | --- | --- | --- |
| Gerull, S.(2013)^31^ | 95 | 63(2years)  48(5years) | 42(2years)  28(5years) | 17(5years) | aGVHD(1)8  aGVHD(2)33  aGVHD(3)9  aGVHD(4)5  Total(cGVHD)87  ext. cGVHD:44  lim. cGVHD:6 | 50 | 48  30 deaths being related to relapse  18 patients died of TRM |
| Giaccone, L. (2011)^32^ | 58 | Median OS  Was not reached | EFS was  39 months in the 58 patients | NA | aGVHD(2-4)23  Total(aGVHD)58  Total(cGVHD):55  ext. cGVHD:32  lim. cGVHD:9 | 30 | 24  13 disease progression,  9 TRM , 2 lung cancer. |
| Karlin, L.(2011)^33^ | 23 | 14(2years) | NA | 4(1years) | aGVHD(1)3  aGVHD(2)12  aGVHD(3)4  Total(cGVHD)19  ext. cGVHD:10  lim. cGVHD:2 | NA | NA |
| Krishnan, A(2011)^34^ | Standard risk :189  High risk:37 | Standard risk  172(1years)  161(2years)  146(3years)  High risk:  22(3years) | Standard risk:  81(3years)  High risk:  15(3years) | Standard risk:  21(3years)  High risk:  8(3years) | Standard risk  aGVHD(2-4)49  aGVHD(3-4)17  cGVHD:89(1years)  cGVHD:112(2years) | Standard risk:  87(3years)  High risk:  14(3years) | Standard risk:60  Relapse/progression22  Organ failure 12, infection10,GVHD8, idiopathic pneumonia 3 , acute respiratory distress 2, graft failure 1, secondary malignancy1, suicide 1 |
| Kroger, N.(2013)^35^ | 73 | 39(5years) | 21(5years) | 17(1years) | aGVHD(1)12  aGVHD(2)20  aGVHD(3)9  aGVHD(4)1 | 31(5years) | NA |

| Author(years) | N | OS | DFS/EFS/PFS | TRM/NRM | GVHD | RR | Death and Death cause |
| --- | --- | --- | --- | --- | --- | --- | --- |
| Kroger, N. (2010)^36^ | 48 | 12(5years) | 10(5years) | 12(1years) | aGVHD(1)11  aGVHD(2)9  aGVHD(3)2  aGVHD(4)1  Total(cGVHD)38  ext. cGVHD:2  lim. cGVHD:11 | 13(1years)  26(3years) | NA |
| Zabelina, T.(2013)^37^ | 33 | 26(3years) | 17(3years) | 2(1years) | aGVHD(1)11  aGVHD(2)1  aGVHD(3)7  aGVHD(4)3  ext. cGVHD:4  lim. cGVHD:7 | 14(3years) | NA |
| Kumar, S.(2011)^38^ | 1207  1989-1994:343 1995-2000:376  2001-2005:488 | 1989-1994 :  172(1years)  123(3years)  103(5years)  1995-2000:  184(1years)  135(3years)  113(5years)  2001-2005:  307(1years)  205(3years)  142(5years) | 1989-1994 :  127(1years)  82(3years)  72(5years)  1995-2000 :  147(1years)  105(3years)  83(5years)  2001-2005:  224(1years)  122(3years)  68(5years) | 1989-1994 :  123(1years)  137(3years)  137(5years)  1995-2000 :  158(1years)  169(3years)  173(5years)  2001-2005:  107(1years)  132(3years)  137(5years) | 1989-1994:  Total(aGVHD):342  aGVHD(2-4):99  Total(cGVHD)339  cGVHD(1years)108  cGVHD(3years)112  cGVHD(5years)112  1995-2000:  Total(aGVHD):376  aGVHD(2-4):128  Total(cGVHD)373  cGVHD(1years)104  cGVHD(3years)112  cGVHD(5years)112  2001-2005:  Total(aGVHD):487  aGVHD(2-4):170  Total(cGVHD):475  cGVHD(1years)219  cGVHD(3years)233  cGVHD(5years)242 | 1989-1994 :  93(1years)  123(3years)  134(5years)  1995-2000 :  71(1years)  105(3years)  120(5years)  2001-2005:  161(1years)  234(3years)  283(5years) | 1989-1994 : 249  Primary disease(MM)111 /Infection39/GVHD15 /Pneumonitis17/Organ failure 19/Second malignancy1 /Graft failure3/Hemorrhage 6/Missing 38  1995-2000 :274  Primary disease (MM) 80 /Infection44/ GVHD 22 /Pneumonitis14/Organ failure 37/Second malignancy5/Accidental death 3 /Graft failure 1  Hemorrhage 6 / Missing 62  2001-2005: 273  Primary disease (MM) 112 /Infection48/ GVHD 18 /Pneumonitis6/Organ failure 25 /Second malignancy3/ Graft failure1/Hemorrhage1/ Missing59 |
| Lokhorst, Hm(2012)^39^ | 122 | 67(6years) | 34(6years) | 20(6years) | aGVHD(3)3  aGVHD(4)4  Total(aGVHD):80  Total(cGVHD):80  ext. cGVHD:44  lim. cGVHD:7 | 67(6years) | NA |
| Minnema, M. C.(2011) ^40^ | 38 | 3.1 years (range, 0.2–7.2) | 1.4 years (0.1–4.9) | 3(1years)  6(3years) | aGVHD(2-4):9  cGVHD(3years):11  cGVHD(4years):18 | 28 | NA |

| Author(years) | N | OS | DFS/EFS/PFS | TRM/NRM | GVHD | RR | Death and Death cause |
| --- | --- | --- | --- | --- | --- | --- | --- |
| Nishihori, T.(2013)^41^ | 22 | 17(2years) | 16(2years) | 2(6 months) | aGVHD(2-4):10  cGVHD:10 | NA | 5  4 patients died of GVHD-related complications, 1 died of disease progression |
| Nivison-Smith, I.(2011)^42^ | 95 | 38(5years) | 26(5years) | 10(100days)  18(1years)  19(2years)  21(3years) | aGVHD(2-4)23  cGVHD(1years):45  cGVHD(2years):48  ext. cGVHD:29  Total(cGVHD):84 | 29(1years)  41(2years) | 54  Relapse/progression 28; GVHD 9 ;Infection 7 ;Organ failure3;Graft failure/rejection 2 ;  Hemorrhage 1 ;  Interstitial pneumonitis (IP) 1  ;New malignancy 1;Other 2 |
| Osman, K.(2010)^43^ | 20 | 5(3years) | 5(3years) | 5(100days) | aGVHD(1)2  aGVHD(2)1  aGVHD(3)3  aGVHD(4)0  ext. cGVHD:4  lim. cGVHD:3 | NA | NA |
| Passera, R.(2013)^44^ | 196 | NA | NA | 51(1years)  65(5years) | aGVHD(2-4):90  cGVHD:68  Total(cGVHD)134 | 56(1years)  98(5years) | 128  57 died of disease  progression, 71 died of transplant-related  causes |
| Patriarca, F.(2012)^45^ | 68 | 50(1years)  37(2years) | 44(1years)  26(2years) | 12(1years)  15(2years) | aGVHD(2-4):28  Total(cGVHD):53  ext. cGVHD:15  lim. cGVHD:6 | 30 | 36  19 due to disease progression and 17 due to  transplantation-related causes. |
| Pawarode, A.(2016)^46^ | 22 | 13(1years)  6(3years) | 9(1years)  3(3years) | 2(100days)  4(1years)  6(3years) | aGVHD(2-4):9  aGVHD(3-4)5  cGVHD(6months):9  cGVHD(1years):15 | 3(100days)  8(1years)  11(3years) | 15  The causes of death were MM progression in 6 patients (27%), severe organ toxicities in 3 (14%), severe chronic GVHD with/without secondary infection in 3 (14%; 2 GVHDs of gastrointestinal tract and 1 bronchiolitis obliterans), very severe acute GVHD of skin/liver/gastrointestinal tract in 1 HLA-DR mismatched patient (4%), and a late unknown etiology in 1 (4%) |

| Author(years) | N | OS | DFS/EFS/PFS | TRM/NRM | GVHD | RR | Death and Death cause |
| --- | --- | --- | --- | --- | --- | --- | --- |
| Ramasamy,K.(2011)^47^ | 19 | 8(2years) | 7(2years) | 4(2years) | lim. cGVHD:2  aGVHD:0 | NA | NA |
| Roos-Weil, D. (2011)^48^ | 143 | 76(3years) | 47(3years) | 36(2years) | aGVHD(1)22  aGVHD(2-4)44  Total(cGVHD):123  ext. cGVHD:31  lim. cGVHD:22 | NA | NA |
| Rosinol, L.(2015)^49^ | MA:25  RIC:33 | MAC:4(10years)  RIC: 13(5years) | MAC:2(10years)  RIC:7(5years) | MAC:15(any time)  RIC:11(any time) | MAC:  aGVHD(2-4)10  aGVHD(3-4)7  Total(cGVHD)18  ext. cGVHD:2  lim. cGVHD:5  RIC:  aGVHD(2-4)13  aGVHD(3-4)8  Total(cGVHD)27  ext. cGVHD:3  lim. cGVHD:8 | MAC:8  RIC:17 | MAC:15  The causes of death were infectious complications related to GVHD in six patients, infections not related to GVHD in seven cases and veno-oclusive disease and multiorgan failure (one patient each).  RIC:11  The causes of death were infections related to GVHD in nine patients, and 1 pulmonary toxicity and 1 post-transplant lymphoma |
| Rosinol, L.(2008)^50^ | 25 | 15(5years) | 15(5years) | 4 | aGVHD(2-4)8  cGVHD:14  Total(cGVHD):21 | NA | NA |
| Rotta, M.(2009)^51^ | 102 | 65(5years) | 37(5years) | 1(100days)  12(1years)  14(2years)  18(5years) | aGVHD(2)34  aGVHD(3)4  aGVHD(4)5  ext. cGVHD:74 | 51 | 39  Disease progression 20 Nonrelapse-related 19 |

| Author  (years) | N | OS | DFS/EFS/PFS | TRM/NRM | GVHD | RR | Death and Death cause |
| --- | --- | --- | --- | --- | --- | --- | --- |
| Sahebi, F.(2015)^52^ | Auto–allo:517  Early RIC:173 | Auto–allo:  352(3years)  305(5years)  Early RIC:  93(3years)  73(5years) | Auto–allo:  233(3years)  176(5years)  Early RIC:  57(3years)  38(5years) | Auto–allo:  41(1years)  67(3years)  Early RIC:  35(1years)  48(3years) | We can’t ensure the total people of cGVHD and aGVHD. so we excluded this article when we pool estimates of GVHD.  Auto–allo:  aGVHD(1-2)150  aGVHD(3-4)51  ext. cGVHD:111  lim. cGVHD:99  Early RIC: aGVHD(1-2)49  aGVHD(3-4)19  ext. cGVHD:27  lim. cGVHD:26 | Auto–allo:  212(3years)  259(5years)  Early RIC:  67(3years)  80(5years) | Auto–allo:234  Relapse/progression122  Infection63 GVHD14  Organ toxicity8  Secondary malignancy5  Other22  Early RIC:96  Relapse/progression42  Infection28 GVHD6  Organ toxicity2  Secondary malignancy3  Other15 |
| Schilling, G. (2008)^53^ | 101 | NA | NA | 21(1years) | aGVHD(1)13  aGVHD(2)21  aGVHD(3)1  aGVHD(4)4  Total(aGVHD):100  Total(cGVHD):90  cGVHD:22 | 38 | NA |
| Shimoni, A.(2010)^54^ | 50 | 17(7years) | 13(7years) | 13(5years) | aGVHD(1)7  aGVHD(2)11  aGVHD(3)4  aGVHD(4)3  Total(aGVHD):47  Total(cGVHD)40  cGVHD:20 | NA | 34  13 patients died of treatment-related complications and 21 of disease recurrence |
| van Dorp, S.(2007)^55^ | 59  frst-line treatment：36  salvage therapy：23 | 50(2years)  frst-line treatment：  32(2years)  salvage therapy：  18(2years) | NA  frst-line treatment：  25(2years)  salvage therapy：  5(2years) | 7(1years) | aGVHD(1)12  aGVHD(2)12  aGVHD(3)14  aGVHD(4)14  ext. cGVHD:29  lim. cGVHD:3 | 25 | 11  4 from progressive disease and 7 from nonrelapse mortality |
| Smith, E.(2016)^56^ | 44 | 24(2years)  18(4years) | 14(2years)  8(4years) | 8(1years) | aGVHD(2-4)1  cGVHD:0 | NA | NA |
| Costa, L. J.(2009)^57^ | 33 | 40 months | NA | 9(1years) | aGVHD(1-4)18  aGVHD(3-4)11  Total(cGVHD):27  ext. cGVHD:16 | NA | 21  The most common causes of death were progression of MM (48%), respiratory complications (20%), acute GVHD (10%) and infection (10%). |
| Mir, M. A.(2015)^58^ | 66 | 24 (0.1-259)  months | 15.3 (0.3-259) months | 13(100days) | aGVHD(1)10  aGVHD(2-4)29  ext. cGVHD:24  lim. cGVHD:8 | 43 | 61  MM 26  Infection 13  GVHD 8  Organ failure 14 |
| Nair, A. P.(2017)^59^ | Upfront:29  Deferred:30 | Upfront:  21(3years)  17(5years)  Deferred:  17(3years)  14(5years) | Upfront:  15(3years)  12(5years)  Deferred:  12(3years)  9(5years) | Upfront:6  Deferred:2 | Upfront:  aGVHD(2-4)7  ext. cGVHD:16  Deferred:  aGVHD(2-4)8  ext. cGVHD:19 | NA | NA |
| Kawamura, K.(2016)^60^ | 89 | 65(3years)  48(6years) | NA | NA | NA | NA | NA |
| Kikuchi, T.(2015)^61^ | 23 | 9(5years) | 1(5years) | 2(3years) | aGVHD(2)6  aGVHD(3)5  aGVHD(4)1  Total(cGVHD)21  ext. cGVHD:7  lim. cGVHD:6 | 21 | 16  The primary causes of death in the remaining patients included relapsed or progressed multiple myeloma in 11 and suicide in 1 |

**When the total number of the specific subgroup is different from the given total number of the first grid, we would provide the total number of the specific subgroup in its’ grid.**

**GVHD: graft-versus-host disease; aGVHD: acute GVHD; ext.cGVHD: extensive chronic GVHD; lim. cGVHD: limited chronic GVHD ; OS: overall survival ;PFS: progression-free survival; DFS: disease-free survival ;EFS: event-free survival; TRM: treatment-related mortality ;NRM: non-relapse mortality ; RR: relapse rate ;**

1 Majolino, I. *et al.* Reduced intensity conditioning with thiotepa, fludarabine, and melphalan is effective in advanced multiple myeloma. *Leukemia and Lymphoma* **48**, 759-766 (2007).

2 Novitzky, N., Thomas, V. & du Toit, C. Prevention of graft vs. host disease with alemtuzumab 'in the bag' decreases early toxicity of stem cell transplantation and in multiple myeloma is associated with improved long-term outcome. *Cytotherapy* **10**, 45-53, doi:10.1080/14653240701732771 (2008).

3 Ringden, O. *et al.* Effect of acute and chronic GVHD on relapse and survival after reduced-intensity conditioning allogeneic transplantation for myeloma. *Bone marrow transplantation* **47**, 831-837, doi:10.1038/bmt.2011.192 (2012).

4 Sahebi, F. *et al.* Late relapses following reduced intensity allogeneic transplantation in patients with multiple myeloma: a long-term follow-up study. *British journal of haematology* **160**, 199-206, doi:10.1111/bjh.12123 (2013).

5 Schmidt-Hieber, M. *et al.* Reduced-toxicity conditioning with fludarabine and treosulfan prior to allogeneic stem cell transplantation in multiple myeloma. *Bone marrow transplantation* **39**, 389-396, doi:10.1038/sj.bmt.1705605 (2007).

6 Ahmad, I. *et al.* Favorable long-term outcome of patients with multiple myeloma using a frontline tandem approach with autologous and non-myeloablative allogeneic transplantation. *Bone marrow transplantation* **51**, 529-535, doi:10.1038/bmt.2015.319 (2016).

7 Auner, H. W. *et al.* Reduced intensity-conditioned allogeneic stem cell transplantation for multiple myeloma relapsing or progressing after autologous transplantation: A study by the European group for blood and marrow transplantation. *Bone Marrow Transplantation* **48**, 1395-1400 (2013).

8 Bashir, Q. *et al.* Predictors of prolonged survival after allogeneic hematopoietic stem cell transplantation for multiple myeloma. *American journal of hematology* **87**, 272-276, doi:10.1002/ajh.22273 (2012).

9 Beaussant, Y. *et al.* Hematopoietic Stem Cell Transplantation in Multiple Myeloma: A Retrospective Study of the Societe Francaise de Greffe de Moelle et de Therapie Cellulaire (SFGM-TC). *Biology of Blood and Marrow Transplantation* **21**, 1452-1459 (2015).

10 Bjorkstrand, B. *et al.* Tandem autologous/reduced-intensity conditioning allogeneic stem-cell transplantation versus autologous transplantation in myeloma: long-term follow-up. *Journal of clinical oncology : official journal of the American Society of Clinical Oncology* **29**, 3016-3022, doi:10.1200/jco.2010.32.7312 (2011).

11 Bruno, B. *et al.* A comparison of allografting with autografting for newly diagnosed myeloma. *The New England journal of medicine* **356**, 1110-1120, doi:10.1056/NEJMoa065464 (2007).

12 Bruno, B. *et al.* Unrelated donor haematopoietic cell transplantation after non-myeloablative conditioning for patients with high-risk multiple myeloma. *European journal of haematology* **78**, 330-337, doi:10.1111/j.1600-0609.2007.00816.x (2007).

13 Crawley, C. *et al.* Reduced-intensity conditioning for myeloma: lower nonrelapse mortality but higher relapse rates compared with myeloablative conditioning. *Blood* **109**, 3588-3594, doi:10.1182/blood-2006-07-036848 (2007).

14 Hong, J. Y. *et al.* Feasibility of second hematopoietic stem cell transplantation using reduced-intensity conditioning with fludarabine and melphalan after a failed autologous hematopoietic stem cell transplantation. *Transplantation Proceedings* **42**, 3723-3728 (2010).

15 Warlick, E. D. *et al.* Reduced-intensity conditioning followed by related allografts in hematologic malignancies: long-term outcomes most successful in indolent and aggressive Non-Hodgkin lymphomas. *Biology of Blood and Marrow Transplantation* **17**, 1025-1032 (2011).

16 Jamshed, S. *et al.* EPOCH-F: A novel salvage regimen for multiple myeloma before reduced-intensity allogeneic hematopoietic SCT. *Bone marrow transplantation* **46**, 676-681 (2011). <<http://onlinelibrary.wiley.com/o/cochrane/clcentral/articles/298/CN-00891298/frame.html>

<http://www.nature.com/bmt/journal/v46/n5/pdf/bmt2010173a.pdf>>.

17 Kroger, N. *et al.* Long-term follow-up of an intensified myeloablative conditioning regimen with in vivo T cell depletion followed by allografting in patients with advanced multiple myeloma. *Biology of blood and marrow transplantation : journal of the American Society for Blood and Marrow Transplantation* **16**, 861-864, doi:10.1016/j.bbmt.2010.01.018 (2010).

18 Bruno, B. *et al.* Nonmyeloablative allografting for newly diagnosed multiple myeloma: the experience of the Gruppo Italiano Trapianti di Midollo. *Blood* **113**, 3375-3382, doi:10.1182/blood-2008-07-167379 (2009).

19 Caballero-Velazquez, T. *et al.* Phase II clinical trial for the evaluation of bortezomib within the reduced intensity conditioning regimen (RIC) and post-allogeneic transplantation for high-risk myeloma patients. *British journal of haematology* **162**, 474-482, doi:10.1111/bjh.12410 (2013).

20 de Lavallade, H. *et al.* Reduced-intensity conditioning allogeneic SCT as salvage treatment for relapsed multiple myeloma. *Bone marrow transplantation* **41**, 953-960, doi:10.1038/bmt.2008.22 (2008).

21 Dhakal, B. *et al.* Allogeneic Hematopoietic Cell Transplantation in Multiple Myeloma: Impact of Disease Risk and Post Allograft Minimal Residual Disease on Survival. *Clinical lymphoma, myeloma & leukemia* **16**, 379-386 (2016). <<http://onlinelibrary.wiley.com/o/cochrane/clcentral/articles/587/CN-01165587/frame.html>

<http://ac.els-cdn.com/S2152265016300131/1-s2.0-S2152265016300131-main.pdf?_tid=df5e57e2-37a3-11e7-b820-00000aab0f6b&acdnat=1494656442_713d55cb5b643a3fc30bb7fec09435f7>>.

22 Efebera, Y. A. *et al.* Reduced-Intensity Allogeneic Hematopoietic Stem Cell Transplantation for Relapsed Multiple Myeloma. *Biology of Blood and Marrow Transplantation* **16**, 1122-1129 (2010).

23 El-Cheikh, J. *et al.* Comparable outcomes between unrelated and related donors after reduced-intensity conditioning allogeneic hematopoietic stem cell transplantation in patients with high-risk multiple myeloma. *European Journal of Haematology* **88**, 497-503 (2012).

24 El-Cheikh, J. *et al.* Long-term outcome after allogeneic stem-cell transplantation with reduced-intensity conditioning in patients with multiple myeloma. *American Journal of Hematology* **88**, 370-374 (2013).

25 Fabre, C. *et al.* Younger donor's age and upfront tandem are two independent prognostic factors for survival in multiple myeloma patients treated by tandem autologous-allogeneic stem cell transplantation: a retrospective study from the Societe Francaise de Greffe de Moelle et de Therapie Cellulaire (SFGM-TC). *Haematologica* **97**, 482-490, doi:10.3324/haematol.2011.049742 (2012).

26 Franssen, L. E. *et al.* Outcome of allogeneic transplantation in newly diagnosed and relapsed/refractory multiple myeloma: long-term follow-up in a single institution. *European journal of haematology* **97**, 479-488, doi:10.1111/ejh.12758 (2016).

27 Freytes, C. O. *et al.* Second transplants for multiple myeloma relapsing after a previous autotransplant-reduced-intensity allogeneic vs autologous transplantation. *Bone marrow transplantation* **49**, 416-421, doi:10.1038/bmt.2013.187 (2014).

28 Gahrton, G. *et al.* Peripheral blood or bone marrow cells in reduced-intensity or myeloablative conditioning allogeneic HLA identical sibling donor transplantation for multiple myeloma. *Haematologica* **92**, 1513-1518, doi:10.3324/haematol.11353 (2007).

29 Gahrton, G. *et al.* Autologous/reduced-intensity allogeneic stem cell transplantation vs autologous transplantation in multiple myeloma: long-term results of the EBMT-NMAM2000 study. *Blood* **121**, 5055-5063, doi:10.1182/blood-2012-11-469452 (2013).

30 Georges, G. E. *et al.* Nonmyeloablative unrelated donor hematopoietic cell transplantation to treat patients with poor-risk, relapsed, or refractory multiple myeloma. *Biology of blood and marrow transplantation : journal of the American Society for Blood and Marrow Transplantation* **13**, 423-432, doi:10.1016/j.bbmt.2006.11.011 (2007).

31 Gerull, S. *et al.* Allo-SCT for multiple myeloma in the era of novel agents: a retrospective study on behalf of Swiss Blood SCT. *Bone marrow transplantation* **48**, 408-413, doi:10.1038/bmt.2012.167 (2013).

32 Giaccone, L. *et al.* Long-term follow-up of a comparison of nonmyeloablative allografting with autografting for newly diagnosed myeloma. *Blood* **117**, 6721-6727, doi:10.1182/blood-2011-03-339945 (2011).

33 Karlin, L. *et al.* Tandem autologous non-myeloablative allogeneic transplantation in patients with multiple myeloma relapsing after a first high dose therapy. *Bone marrow transplantation* **46**, 250-256, doi:10.1038/bmt.2010.90 (2011).

34 Krishnan, A. *et al.* Autologous haemopoietic stem-cell transplantation followed by allogeneic or autologous haemopoietic stem-cell transplantation in patients with multiple myeloma (BMT CTN 0102): a phase 3 biological assignment trial. *The Lancet. Oncology* **12**, 1195-1203 (2011). <<http://onlinelibrary.wiley.com/o/cochrane/clcentral/articles/504/CN-00805504/frame.html>

<http://ac.els-cdn.com/S1470204511702431/1-s2.0-S1470204511702431-main.pdf?_tid=190c0afc-37a4-11e7-bb52-00000aacb35e&acdnat=1494656539_84eaeddfc36ab4ba83517a7e12017653>>.

35 Kroger, N. *et al.* Impact of high-risk cytogenetics and achievement of molecular remission on long-term freedom from disease after autologous-allogeneic tandem transplantation in patients with multiple myeloma. *Biology of blood and marrow transplantation : journal of the American Society for Blood and Marrow Transplantation* **19**, 398-404, doi:10.1016/j.bbmt.2012.10.008 (2013).

36 Kroger, N. *et al.* Unrelated stem cell transplantation after reduced intensity conditioning for patients with multiple myeloma relapsing after autologous transplantation. *British journal of haematology* **148**, 323-331, doi:10.1111/j.1365-2141.2009.07984.x (2010).

37 Zabelina, T. *et al.* Toxicity-reduced, myeloablative allograft followed by lenalidomide maintenance as salvage therapy for refractory/relapsed myeloma patients. *Bone marrow transplantation* **48**, 403-407, doi:10.1038/bmt.2012.142 (2013).

38 Kumar, S. *et al.* Trends in allogeneic stem cell transplantation for multiple myeloma: a CIBMTR analysis. *Blood* **118**, 1979-1988, doi:10.1182/blood-2011-02-337329 (2011).

39 Lokhorst, H. *et al.* Donor versus no-donor comparison of newly diagnosed myeloma patients included in the HOVON-50 multiple myeloma study. *Blood* **119**, 6219-6225; quiz 6399 (2012). <<http://onlinelibrary.wiley.com/o/cochrane/clcentral/articles/088/CN-00970088/frame.html>

<http://www.bloodjournal.org/content/bloodjournal/119/26/6219.full.pdf>>.

40 Minnema, M. C. *et al.* Prognostic factors and outcome in relapsed multiple myeloma after nonmyeloablative allo-SCT: a single center experience. *Bone marrow transplantation* **46**, 244-249, doi:10.1038/bmt.2010.101 (2011).

41 Nishihori, T. *et al.* Allogeneic hematopoietic cell transplantation for consolidation of VGPR or CR for newly diagnosed multiple myeloma. *Bone marrow transplantation* **48**, 1179-1184, doi:10.1038/bmt.2013.37 (2013).

42 Nivison-Smith, I. *et al.* Allogeneic hematopoietic cell transplant for multiple myeloma using reduced intensity conditioning therapy, 1998-2006: factors associated with improved survival outcome. *Leukemia & lymphoma* **52**, 1727-1735, doi:10.3109/10428194.2011.582201 (2011).

43 Osman, K. *et al.* Non-myeloablative conditioning and allogeneic transplantation for multiple myeloma. *American journal of hematology* **85**, 249-254, doi:10.1002/ajh.21633 (2010).

44 Passera, R. *et al.* Allogeneic hematopoietic cell transplantation from unrelated donors in multiple myeloma: study from the Italian Bone Marrow Donor Registry. *Biology of blood and marrow transplantation : journal of the American Society for Blood and Marrow Transplantation* **19**, 940-948, doi:10.1016/j.bbmt.2013.03.012 (2013).

45 Patriarca, F. *et al.* Allogeneic stem cell transplantation in multiple myeloma relapsed after autograft: A multicenter retrospective study based on donor availability. *Biology of Blood and Marrow Transplantation* **18**, 617-626 (2012).

46 Pawarode, A. *et al.* Reducing Treatment-Related Mortality Did Not Improve Outcomes of Allogeneic Myeloablative Hematopoietic Cell Transplantation for High-Risk Multiple Myeloma: A University of Michigan Prospective Series. *Biology of Blood and Marrow Transplantation* **22**, 54-60 (2016).

47 Ramasamy, K. *et al.* Alemtuzumab-based reduced-intensity conditioning allogeneic transplantation for myeloma and plasma cell leukemia - a single-institution experience. *Clinical lymphoma, myeloma & leukemia* **11**, 242-245, doi:10.1016/j.clml.2011.03.004 (2011).

48 Roos-Weil, D. *et al.* Impact of genetic abnormalities after allogeneic stem cell transplantation in multiple myeloma: a report of the Societe Francaise de Greffe de Moelle et de Therapie Cellulaire. *Haematologica* **96**, 1504-1511, doi:10.3324/haematol.2011.042713 (2011).

49 Rosinol, L. *et al.* Allogeneic hematopoietic SCT in multiple myeloma: Long-term results from a single institution. *Bone Marrow Transplantation* **50**, 658-662 (2015).

50 Rosinol, L. *et al.* A prospective PETHEMA study of tandem autologous transplantation versus autograft followed by reduced-intensity conditioning allogeneic transplantation in newly diagnosed multiple myeloma. *Blood* **112**, 3591-3593, doi:10.1182/blood-2008-02-141598 (2008).

51 Rotta, M. *et al.* Long-term outcome of patients with multiple myeloma after autologous hematopoietic cell transplantation and nonmyeloablative allografting. *Blood* **113**, 3383-3391, doi:10.1182/blood-2008-07-170746 (2009).

52 Sahebi, F. *et al.* Comparison of upfront tandem autologous-allogeneic transplantation versus reduced intensity allogeneic transplantation for multiple myeloma. *Bone marrow transplantation* **50**, 802-807, doi:10.1038/bmt.2015.45 (2015).

53 Schilling, G. *et al.* Impact of genetic abnormalities on survival after allogeneic hematopoietic stem cell transplantation in multiple myeloma. *Leukemia* **22**, 1250-1255, doi:10.1038/leu.2008.88 (2008).

54 Shimoni, A. *et al.* Allogenic hematopoietic stem-cell transplantation with reduced-intensity conditioning in patients with refractory and recurrent multiple myeloma: long-term follow-up. *Cancer* **116**, 3621-3630, doi:10.1002/cncr.25228 (2010).

55 van Dorp, S. *et al.* Single-centre experience with nonmyeloablative allogeneic stem cell transplantation in patients with multiple myeloma: Prolonged remissions induced. *Netherlands Journal of Medicine* **65**, 178-184 (2007).

56 Smith, E. *et al.* CD34-Selected Allogeneic Hematopoietic Stem Cell Transplantation for Patients with Relapsed, High-Risk Multiple Myeloma. *Biology of blood and marrow transplantation : journal of the American Society for Blood and Marrow Transplantation* **22**, 258-267, doi:10.1016/j.bbmt.2015.08.025 (2016).

57 Costa, L. J. *et al.* Factors associated with favorable outcome after allogeneic hematopoietic stem cell transplantation for multiple myeloma. *Leukemia & lymphoma* **50**, 781-787, doi:10.1080/10428190902803644 (2009).

58 Mir, M. A. *et al.* Trends and outcomes in allogeneic hematopoietic stem cell transplant for multiple myeloma at Mayo Clinic. *Clinical lymphoma, myeloma & leukemia* **15**, 349-357.e342, doi:10.1016/j.clml.2015.03.016 (2015).

59 Nair, A. P. *et al.* Adverse impact of high donor CD3+ cell dose on outcome following tandem auto-NMA allogeneic transplantation for high-risk myeloma. *Bone Marrow Transplantation.* **20** (2017).

60 Kawamura, K. *et al.* Tandem autologous versus autologous/allogeneic transplantation for multiple myeloma: propensity score analysis. *Leukemia and Lymphoma* **57**, 2077-2083 (2016).

61 Kikuchi, T. *et al.* Outcome of reduced-intensity allogeneic hematopoietic stem cell transplantation for multiple myeloma. *International Journal of Hematology* **102**, 670-677 (2015).
