# Supplementary material for: Allogeneic stem-cell transplantation for multiple myeloma: a systematic review and meta-analysis from 2007 to 2017
Source: Cancer Cell Int. 2018 Apr 23;18:62. doi: 10.1186/s12935-018-0553-8 (PMC5913895; doi:10.1186/s12935-018-0553-8)
Supplement: Supplementary file 3 — Additional file 3: Table S3. Quality assessment of individual clinical trials. [file 12935_2018_553_MOESM3_ESM.docx]

| Study | Majolino, I.  (2007)^1^ | Novitzky, N.  (2008)^2^ | Ringden, O.  (2012)^3^ | Sahebi, F.  (2013)^4^ | Kikuchi, T.  (2015)^5^ |
| --- | --- | --- | --- | --- | --- |
| A | + | + | + | + | + |
| B | + | + | + | + | + |
| C | + | + | + | + | + |
| D | + | + | + | - | + |
| E | - | - | + | + | + |
| Total | 4 | 4 | 5 | 4 | 5 |

**A: conditioning regimen; B: stem cell source; C:donor; D: GvHD prophylaxis regimen; E: disease status before allo-SCT;**

**+:yes; -:no ; ？：unclear**

| Study | Schmidt-Hieber, M.(2007)^6^ | Ahmad, I.  （2016）^7^ | Auner,H.W.  (2013)^8^ | Bashir, Q.  (2012)^9^ | Beaussant, Y.(2015)^10^ |
| --- | --- | --- | --- | --- | --- |
| A | + | + | + | + | + |
| B | + | - | + | + | + |
| C | + | + | + | + | + |
| D | + | + | + | + | + |
| E | + | + | + | + | + |
| Total | 5 | 4 | 5 | 5 | 5 |

| Study | Bjorkstrand, B.(2011)^11^ | Bruno, B  (2007)^12^ | Sorasio, R.  (2007)^13^ | Crawley, C.  （2007）^14^ | Hong, J. Y.  (2010)^15^ |
| --- | --- | --- | --- | --- | --- |
| A | + | + | + | + | + |
| B | - | + | + | + | - |
| C | + | + | + | + | + |
| D | + | - | + | + | + |
| E | + | + | + | + | + |
| Total | 4 | 4 | 5 | 5 | 4 |

| Study | Warlick, E. D.(2011)^16^ | Jamshed,S (2011)^17^ | Einsele, H. (2010)^18^ | Bruno, B.  (2009)^19^ | Caballero-Velazquez, T.(2013)^20^ |
| --- | --- | --- | --- | --- | --- |
| A | + | + | + | + | + |
| B | - | - | + | + | + |
| C | + | + | + | + | + |
| D | + | + | + | - | + |
| E | - | - | + | + | + |
| Total | 3 | 3 | 5 | 4 | 5 |

| Study | de Lavallade, H.(2008)^21^ | Dhakal, B  (2016)^22^ | Efebera, Y. A.(2010)^23^ | El-Cheikh, J.(2012)^24^ | El-Cheikh, J.(2013)^25^ |
| --- | --- | --- | --- | --- | --- |
| A | + | + | + | + | + |
| B | + | - | + | + | - |
| C | + | + | + | + | + |
| D | + | + | + | + | - |
| E | + | + | + | + | + |
| Total | 5 | 4 | 5 | 5 | 3 |

| Study | Fabre, C  （2012）^26^ | Franssen, L. E.(2016)^27^ | Freytes, C. O.(2014)^28^ | Gahrton, G.  (2007)^29^ | Gahrton, G.  (2013)^30^ |
| --- | --- | --- | --- | --- | --- |
| A | + | + | + | + | + |
| B | + | + | + | + | - |
| C | + | + | + | + | + |
| D | + | - | + | - | + |
| E | + | + | + | + | - |
| Total | 5 | 4 | 5 | 4 | 3 |

| Study | Georges, G. E.（2007）^31^ | Gerull, S.  (2013)^32^ | Giaccone, L.（2011）^33^ | Karlin, L.  (2011)^34^ | Kawamura, K.(2016)^35^ |
| --- | --- | --- | --- | --- | --- |
| A | + | + | + | + | - |
| B | + | + | + | + | + |
| C | + | + | + | + | + |
| D | + | - | - | + | - |
| E | + | + | + | + | + |
| Total | 5 | 4 | 4 | 5 | 3 |

| Study | Krishnan, A  (2011)^36^ | Kroger, N.  (2013)^37^ | Kroger, N (2010)^38^ | Zabelina, T.(2013)^39^ | Kumar, S.  (2011)^40^ |
| --- | --- | --- | --- | --- | --- |
| A | + | + | + | + | + |
| B | + | + | + | + | + |
| C | + | + | + | + | + |
| D | + | + | + | + | + |
| E | + | + | + | + | - |
| Total | 5 | 5 | 5 | 5 | 4 |

| Study | Lokhorst, Hm(2012)^41^ | Minnema, M. C.(2011)^42^ | Nishihori, T.(2013)^43^ | Nivison-Smith, I.  (2011)^44^ | Nair, A. P.  (2017)^45^ |
| --- | --- | --- | --- | --- | --- |
| A | + | + | + | + | + |
| B | - | - | + | + | + |
| C | + | + | + | + | + |
| D | + | + | + | + | + |
| E | + | + | + | + | - |
| Total | 4 | 4 | 5 | 5 | 4 |

| Study | Osman, K.(2010)^46^ | Passera, R.  (2013)^47^ | Patriarca, F.(2012)^48^ | Pawarode, A.(2016)^49^ | Ramasamy, K.(2011)^50^ |
| --- | --- | --- | --- | --- | --- |
| A | + | + | + | + | + |
| B | + | + | + | + | + |
| C | + | + | + | + | + |
| D | + | + | + | + | + |
| E | + | + | - | - | + |
| Total | 5 | 5 | 4 | 4 | 5 |

| Study | Roos-Weil, D.（2011）^51^ | Rosinol, L.  (2015)^52^ | Rosinol, L.  (2008)^53^ | Rotta, M.  (2009)^54^ | Sahebi, F.  (2015)^55^ |
| --- | --- | --- | --- | --- | --- |
| A | - | + | + | + | + |
| B | + | + | - | + | + |
| C | + | + | + | + | + |
| D | - | + | + | + | - |
| E | + | - | - | + | + |
| Total | 3 | 4 | 3 | 5 | 4 |

| Study | Schilling, G.（2008）^56^ | Shimoni, A.  (2010)^57^ | van Dorp, S.(2007)^58^ | Smith, E.  (2016)^59^ | Costa, L. J.(2009)^60^ |
| --- | --- | --- | --- | --- | --- |
| A | + | + | + | + | + |
| B | + | + | - | + | + |
| C | + | + | + | + | + |
| D | - | + | + | - | + |
| E | + | + | + | + | + |
| Total | 4 | 5 | 4 | 4 | 5 |

| Study | Mir, M. A.  (2015)^61^ |
| --- | --- |
| A | + |
| B | - |
| C | + |
| D | + |
| E | + |
| Total | 4 |

1 Majolino, I. *et al.* Reduced intensity conditioning with thiotepa, fludarabine, and melphalan is effective in advanced multiple myeloma. *Leukemia and Lymphoma* **48**, 759-766 (2007).

2 Novitzky, N., Thomas, V. & du Toit, C. Prevention of graft vs. host disease with alemtuzumab 'in the bag' decreases early toxicity of stem cell transplantation and in multiple myeloma is associated with improved long-term outcome. *Cytotherapy* **10**, 45-53, doi:10.1080/14653240701732771 (2008).

3 Ringden, O. *et al.* Effect of acute and chronic GVHD on relapse and survival after reduced-intensity conditioning allogeneic transplantation for myeloma. *Bone marrow transplantation* **47**, 831-837, doi:10.1038/bmt.2011.192 (2012).

4 Sahebi, F. *et al.* Late relapses following reduced intensity allogeneic transplantation in patients with multiple myeloma: a long-term follow-up study. *British journal of haematology* **160**, 199-206, doi:10.1111/bjh.12123 (2013).

5 Kikuchi, T. *et al.* Outcome of reduced-intensity allogeneic hematopoietic stem cell transplantation for multiple myeloma. *International Journal of Hematology* **102**, 670-677 (2015).

6 Schmidt-Hieber, M. *et al.* Reduced-toxicity conditioning with fludarabine and treosulfan prior to allogeneic stem cell transplantation in multiple myeloma. *Bone marrow transplantation* **39**, 389-396, doi:10.1038/sj.bmt.1705605 (2007).

7 Ahmad, I. *et al.* Favorable long-term outcome of patients with multiple myeloma using a frontline tandem approach with autologous and non-myeloablative allogeneic transplantation. *Bone marrow transplantation* **51**, 529-535, doi:10.1038/bmt.2015.319 (2016).

8 Auner, H. W. *et al.* Reduced intensity-conditioned allogeneic stem cell transplantation for multiple myeloma relapsing or progressing after autologous transplantation: A study by the European group for blood and marrow transplantation. *Bone Marrow Transplantation* **48**, 1395-1400 (2013).

9 Bashir, Q. *et al.* Predictors of prolonged survival after allogeneic hematopoietic stem cell transplantation for multiple myeloma. *American journal of hematology* **87**, 272-276, doi:10.1002/ajh.22273 (2012).

10 Beaussant, Y. *et al.* Hematopoietic Stem Cell Transplantation in Multiple Myeloma: A Retrospective Study of the Societe Francaise de Greffe de Moelle et de Therapie Cellulaire (SFGM-TC). *Biology of Blood and Marrow Transplantation* **21**, 1452-1459 (2015).

11 Bjorkstrand, B. *et al.* Tandem autologous/reduced-intensity conditioning allogeneic stem-cell transplantation versus autologous transplantation in myeloma: long-term follow-up. *Journal of clinical oncology : official journal of the American Society of Clinical Oncology* **29**, 3016-3022, doi:10.1200/jco.2010.32.7312 (2011).

12 Bruno, B. *et al.* A comparison of allografting with autografting for newly diagnosed myeloma. *The New England journal of medicine* **356**, 1110-1120, doi:10.1056/NEJMoa065464 (2007).

13 Bruno, B. *et al.* Unrelated donor haematopoietic cell transplantation after non-myeloablative conditioning for patients with high-risk multiple myeloma. *European journal of haematology* **78**, 330-337, doi:10.1111/j.1600-0609.2007.00816.x (2007).

14 Crawley, C. *et al.* Reduced-intensity conditioning for myeloma: lower nonrelapse mortality but higher relapse rates compared with myeloablative conditioning. *Blood* **109**, 3588-3594, doi:10.1182/blood-2006-07-036848 (2007).

15 Hong, J. Y. *et al.* Feasibility of second hematopoietic stem cell transplantation using reduced-intensity conditioning with fludarabine and melphalan after a failed autologous hematopoietic stem cell transplantation. *Transplantation Proceedings* **42**, 3723-3728 (2010).

16 Warlick, E. D. *et al.* Reduced-intensity conditioning followed by related allografts in hematologic malignancies: long-term outcomes most successful in indolent and aggressive Non-Hodgkin lymphomas. *Biology of Blood and Marrow Transplantation* **17**, 1025-1032 (2011).

17 Jamshed, S. *et al.* EPOCH-F: A novel salvage regimen for multiple myeloma before reduced-intensity allogeneic hematopoietic SCT. *Bone marrow transplantation* **46**, 676-681 (2011). <<http://onlinelibrary.wiley.com/o/cochrane/clcentral/articles/298/CN-00891298/frame.html>

<http://www.nature.com/bmt/journal/v46/n5/pdf/bmt2010173a.pdf>>.

18 Kroger, N. *et al.* Long-term follow-up of an intensified myeloablative conditioning regimen with in vivo T cell depletion followed by allografting in patients with advanced multiple myeloma. *Biology of blood and marrow transplantation : journal of the American Society for Blood and Marrow Transplantation* **16**, 861-864, doi:10.1016/j.bbmt.2010.01.018 (2010).

19 Bruno, B. *et al.* Nonmyeloablative allografting for newly diagnosed multiple myeloma: the experience of the Gruppo Italiano Trapianti di Midollo. *Blood* **113**, 3375-3382, doi:10.1182/blood-2008-07-167379 (2009).

20 Caballero-Velazquez, T. *et al.* Phase II clinical trial for the evaluation of bortezomib within the reduced intensity conditioning regimen (RIC) and post-allogeneic transplantation for high-risk myeloma patients. *British journal of haematology* **162**, 474-482, doi:10.1111/bjh.12410 (2013).

21 de Lavallade, H. *et al.* Reduced-intensity conditioning allogeneic SCT as salvage treatment for relapsed multiple myeloma. *Bone marrow transplantation* **41**, 953-960, doi:10.1038/bmt.2008.22 (2008).

22 Dhakal, B. *et al.* Allogeneic Hematopoietic Cell Transplantation in Multiple Myeloma: Impact of Disease Risk and Post Allograft Minimal Residual Disease on Survival. *Clinical lymphoma, myeloma & leukemia* **16**, 379-386 (2016). <<http://onlinelibrary.wiley.com/o/cochrane/clcentral/articles/587/CN-01165587/frame.html>

<http://ac.els-cdn.com/S2152265016300131/1-s2.0-S2152265016300131-main.pdf?_tid=df5e57e2-37a3-11e7-b820-00000aab0f6b&acdnat=1494656442_713d55cb5b643a3fc30bb7fec09435f7>>.

23 Efebera, Y. A. *et al.* Reduced-Intensity Allogeneic Hematopoietic Stem Cell Transplantation for Relapsed Multiple Myeloma. *Biology of Blood and Marrow Transplantation* **16**, 1122-1129 (2010).

24 El-Cheikh, J. *et al.* Comparable outcomes between unrelated and related donors after reduced-intensity conditioning allogeneic hematopoietic stem cell transplantation in patients with high-risk multiple myeloma. *European Journal of Haematology* **88**, 497-503 (2012).

25 El-Cheikh, J. *et al.* Long-term outcome after allogeneic stem-cell transplantation with reduced-intensity conditioning in patients with multiple myeloma. *American Journal of Hematology* **88**, 370-374 (2013).

26 Fabre, C. *et al.* Younger donor's age and upfront tandem are two independent prognostic factors for survival in multiple myeloma patients treated by tandem autologous-allogeneic stem cell transplantation: a retrospective study from the Societe Francaise de Greffe de Moelle et de Therapie Cellulaire (SFGM-TC). *Haematologica* **97**, 482-490, doi:10.3324/haematol.2011.049742 (2012).

27 Franssen, L. E. *et al.* Outcome of allogeneic transplantation in newly diagnosed and relapsed/refractory multiple myeloma: long-term follow-up in a single institution. *European journal of haematology* **97**, 479-488, doi:10.1111/ejh.12758 (2016).

28 Freytes, C. O. *et al.* Second transplants for multiple myeloma relapsing after a previous autotransplant-reduced-intensity allogeneic vs autologous transplantation. *Bone marrow transplantation* **49**, 416-421, doi:10.1038/bmt.2013.187 (2014).

29 Gahrton, G. *et al.* Peripheral blood or bone marrow cells in reduced-intensity or myeloablative conditioning allogeneic HLA identical sibling donor transplantation for multiple myeloma. *Haematologica* **92**, 1513-1518, doi:10.3324/haematol.11353 (2007).

30 Gahrton, G. *et al.* Autologous/reduced-intensity allogeneic stem cell transplantation vs autologous transplantation in multiple myeloma: long-term results of the EBMT-NMAM2000 study. *Blood* **121**, 5055-5063, doi:10.1182/blood-2012-11-469452 (2013).

31 Georges, G. E. *et al.* Nonmyeloablative unrelated donor hematopoietic cell transplantation to treat patients with poor-risk, relapsed, or refractory multiple myeloma. *Biology of blood and marrow transplantation : journal of the American Society for Blood and Marrow Transplantation* **13**, 423-432, doi:10.1016/j.bbmt.2006.11.011 (2007).

32 Gerull, S. *et al.* Allo-SCT for multiple myeloma in the era of novel agents: a retrospective study on behalf of Swiss Blood SCT. *Bone marrow transplantation* **48**, 408-413, doi:10.1038/bmt.2012.167 (2013).

33 Giaccone, L. *et al.* Long-term follow-up of a comparison of nonmyeloablative allografting with autografting for newly diagnosed myeloma. *Blood* **117**, 6721-6727, doi:10.1182/blood-2011-03-339945 (2011).

34 Karlin, L. *et al.* Tandem autologous non-myeloablative allogeneic transplantation in patients with multiple myeloma relapsing after a first high dose therapy. *Bone marrow transplantation* **46**, 250-256, doi:10.1038/bmt.2010.90 (2011).

35 Kawamura, K. *et al.* Tandem autologous versus autologous/allogeneic transplantation for multiple myeloma: propensity score analysis. *Leukemia and Lymphoma* **57**, 2077-2083 (2016).

36 Krishnan, A. *et al.* Autologous haemopoietic stem-cell transplantation followed by allogeneic or autologous haemopoietic stem-cell transplantation in patients with multiple myeloma (BMT CTN 0102): a phase 3 biological assignment trial. *The Lancet. Oncology* **12**, 1195-1203 (2011). <<http://onlinelibrary.wiley.com/o/cochrane/clcentral/articles/504/CN-00805504/frame.html>

<http://ac.els-cdn.com/S1470204511702431/1-s2.0-S1470204511702431-main.pdf?_tid=190c0afc-37a4-11e7-bb52-00000aacb35e&acdnat=1494656539_84eaeddfc36ab4ba83517a7e12017653>>.

37 Kroger, N. *et al.* Impact of high-risk cytogenetics and achievement of molecular remission on long-term freedom from disease after autologous-allogeneic tandem transplantation in patients with multiple myeloma. *Biology of blood and marrow transplantation : journal of the American Society for Blood and Marrow Transplantation* **19**, 398-404, doi:10.1016/j.bbmt.2012.10.008 (2013).

38 Kroger, N. *et al.* Unrelated stem cell transplantation after reduced intensity conditioning for patients with multiple myeloma relapsing after autologous transplantation. *British journal of haematology* **148**, 323-331, doi:10.1111/j.1365-2141.2009.07984.x (2010).

39 Zabelina, T. *et al.* Toxicity-reduced, myeloablative allograft followed by lenalidomide maintenance as salvage therapy for refractory/relapsed myeloma patients. *Bone marrow transplantation* **48**, 403-407, doi:10.1038/bmt.2012.142 (2013).

40 Kumar, S. *et al.* Trends in allogeneic stem cell transplantation for multiple myeloma: a CIBMTR analysis. *Blood* **118**, 1979-1988, doi:10.1182/blood-2011-02-337329 (2011).

41 Lokhorst, H. *et al.* Donor versus no-donor comparison of newly diagnosed myeloma patients included in the HOVON-50 multiple myeloma study. *Blood* **119**, 6219-6225; quiz 6399 (2012). <<http://onlinelibrary.wiley.com/o/cochrane/clcentral/articles/088/CN-00970088/frame.html>

<http://www.bloodjournal.org/content/bloodjournal/119/26/6219.full.pdf>>.

42 Minnema, M. C. *et al.* Prognostic factors and outcome in relapsed multiple myeloma after nonmyeloablative allo-SCT: a single center experience. *Bone marrow transplantation* **46**, 244-249, doi:10.1038/bmt.2010.101 (2011).

43 Nishihori, T. *et al.* Allogeneic hematopoietic cell transplantation for consolidation of VGPR or CR for newly diagnosed multiple myeloma. *Bone marrow transplantation* **48**, 1179-1184, doi:10.1038/bmt.2013.37 (2013).

44 Nivison-Smith, I. *et al.* Allogeneic hematopoietic cell transplant for multiple myeloma using reduced intensity conditioning therapy, 1998-2006: factors associated with improved survival outcome. *Leukemia & lymphoma* **52**, 1727-1735, doi:10.3109/10428194.2011.582201 (2011).

45 Nair, A. P. *et al.* Adverse impact of high donor CD3+ cell dose on outcome following tandem auto-NMA allogeneic transplantation for high-risk myeloma. *Bone Marrow Transplantation.* **20** (2017).

46 Osman, K. *et al.* Non-myeloablative conditioning and allogeneic transplantation for multiple myeloma. *American journal of hematology* **85**, 249-254, doi:10.1002/ajh.21633 (2010).

47 Passera, R. *et al.* Allogeneic hematopoietic cell transplantation from unrelated donors in multiple myeloma: study from the Italian Bone Marrow Donor Registry. *Biology of blood and marrow transplantation : journal of the American Society for Blood and Marrow Transplantation* **19**, 940-948, doi:10.1016/j.bbmt.2013.03.012 (2013).

48 Patriarca, F. *et al.* Allogeneic stem cell transplantation in multiple myeloma relapsed after autograft: A multicenter retrospective study based on donor availability. *Biology of Blood and Marrow Transplantation* **18**, 617-626 (2012).

49 Pawarode, A. *et al.* Reducing Treatment-Related Mortality Did Not Improve Outcomes of Allogeneic Myeloablative Hematopoietic Cell Transplantation for High-Risk Multiple Myeloma: A University of Michigan Prospective Series. *Biology of Blood and Marrow Transplantation* **22**, 54-60 (2016).

50 Ramasamy, K. *et al.* Alemtuzumab-based reduced-intensity conditioning allogeneic transplantation for myeloma and plasma cell leukemia - a single-institution experience. *Clinical lymphoma, myeloma & leukemia* **11**, 242-245, doi:10.1016/j.clml.2011.03.004 (2011).

51 Roos-Weil, D. *et al.* Impact of genetic abnormalities after allogeneic stem cell transplantation in multiple myeloma: a report of the Societe Francaise de Greffe de Moelle et de Therapie Cellulaire. *Haematologica* **96**, 1504-1511, doi:10.3324/haematol.2011.042713 (2011).

52 Rosinol, L. *et al.* Allogeneic hematopoietic SCT in multiple myeloma: Long-term results from a single institution. *Bone Marrow Transplantation* **50**, 658-662 (2015).

53 Rosinol, L. *et al.* A prospective PETHEMA study of tandem autologous transplantation versus autograft followed by reduced-intensity conditioning allogeneic transplantation in newly diagnosed multiple myeloma. *Blood* **112**, 3591-3593, doi:10.1182/blood-2008-02-141598 (2008).

54 Rotta, M. *et al.* Long-term outcome of patients with multiple myeloma after autologous hematopoietic cell transplantation and nonmyeloablative allografting. *Blood* **113**, 3383-3391, doi:10.1182/blood-2008-07-170746 (2009).

55 Sahebi, F. *et al.* Comparison of upfront tandem autologous-allogeneic transplantation versus reduced intensity allogeneic transplantation for multiple myeloma. *Bone marrow transplantation* **50**, 802-807, doi:10.1038/bmt.2015.45 (2015).

56 Schilling, G. *et al.* Impact of genetic abnormalities on survival after allogeneic hematopoietic stem cell transplantation in multiple myeloma. *Leukemia* **22**, 1250-1255, doi:10.1038/leu.2008.88 (2008).

57 Shimoni, A. *et al.* Allogenic hematopoietic stem-cell transplantation with reduced-intensity conditioning in patients with refractory and recurrent multiple myeloma: long-term follow-up. *Cancer* **116**, 3621-3630, doi:10.1002/cncr.25228 (2010).

58 van Dorp, S. *et al.* Single-centre experience with nonmyeloablative allogeneic stem cell transplantation in patients with multiple myeloma: Prolonged remissions induced. *Netherlands Journal of Medicine* **65**, 178-184 (2007).

59 Smith, E. *et al.* CD34-Selected Allogeneic Hematopoietic Stem Cell Transplantation for Patients with Relapsed, High-Risk Multiple Myeloma. *Biology of blood and marrow transplantation : journal of the American Society for Blood and Marrow Transplantation* **22**, 258-267, doi:10.1016/j.bbmt.2015.08.025 (2016).

60 Costa, L. J. *et al.* Factors associated with favorable outcome after allogeneic hematopoietic stem cell transplantation for multiple myeloma. *Leukemia & lymphoma* **50**, 781-787, doi:10.1080/10428190902803644 (2009).

61 Mir, M. A. *et al.* Trends and outcomes in allogeneic hematopoietic stem cell transplant for multiple myeloma at Mayo Clinic. *Clinical lymphoma, myeloma & leukemia* **15**, 349-357.e342, doi:10.1016/j.clml.2015.03.016 (2015).
