# Supplementary material for: Allogeneic stem-cell transplantation for multiple myeloma: a systematic review and meta-analysis from 2007 to 2017
Source: Cancer Cell Int. 2018 Apr 23;18:62. doi: 10.1186/s12935-018-0553-8 (PMC5913895; doi:10.1186/s12935-018-0553-8)
Supplement: Supplementary file 4 — Additional file 4: Table S4. Begg and Egger test of studies included in the meta-analysis. [file 12935_2018_553_MOESM4_ESM.docx]

| **outcomes** | **Begg** | **Egger** |
| --- | --- | --- |
| **OS** |  |  |
| 1 year | 0.174 | 0.624 |
| 2 years | 0.669 | 0.214 |
| 3 years  5 years | 0.592  1.000 | 0.510  0.036 |
| **PFS** |  |  |
| 1 year | 0.548 | 0.393 |
| 2 years | 0.276 | 0.481 |
| 3 years  5 years | 0.921  0.184 | 0.217  0.046 |
| **TRM** |  |  |
| 100 days  1 year | 0.477  0.097 | 0.974  0.730 |
| 2 years  3 years  5 years | 0.640  0.193  0.350 | 0.259  0.367  0.086 |
| **RR** | 0.340 | 0.754 |
| Death  aGVHD  cGVHD  exGVHD  limGVHD | 0.859  0.127  0.976  0.092  0.350 | 0.081  0.265  0.971  0.013  0.431 |
|  |  |  |
